# The determinants of the rarity of nucleic and peptide short sequences in nature

**DOI:** 10.1093/nargab/lqae029

**Published:** 2024-04-04

**Authors:** Nikol Chantzi, Manvita Mareboina, Maxwell A Konnaris, Austin Montgomery, Michail Patsakis, Ioannis Mouratidis, Ilias Georgakopoulos-Soares

**Affiliations:** Institute for Personalized Medicine, Department of Biochemistry and Molecular Biology, The Pennsylvania State University College of Medicine, Hershey, PA, 17033, USA; Institute for Personalized Medicine, Department of Biochemistry and Molecular Biology, The Pennsylvania State University College of Medicine, Hershey, PA, 17033, USA; Institute for Personalized Medicine, Department of Biochemistry and Molecular Biology, The Pennsylvania State University College of Medicine, Hershey, PA, 17033, USA; Department of Statistics, Penn State University, University Park, PA, 16802, USA; Huck Institutes of the Life Sciences, Penn State University, University Park, PA, 16802, USA; Institute for Personalized Medicine, Department of Biochemistry and Molecular Biology, The Pennsylvania State University College of Medicine, Hershey, PA, 17033, USA; Institute for Personalized Medicine, Department of Biochemistry and Molecular Biology, The Pennsylvania State University College of Medicine, Hershey, PA, 17033, USA; Institute for Personalized Medicine, Department of Biochemistry and Molecular Biology, The Pennsylvania State University College of Medicine, Hershey, PA, 17033, USA; Huck Institutes of the Life Sciences, Penn State University, University Park, PA, 16802, USA; Institute for Personalized Medicine, Department of Biochemistry and Molecular Biology, The Pennsylvania State University College of Medicine, Hershey, PA, 17033, USA

## Abstract

The prevalence of nucleic and peptide short sequences across organismal genomes and proteomes has not been thoroughly investigated. We examined 45 785 reference genomes and 21 871 reference proteomes, spanning archaea, bacteria, eukaryotes and viruses to calculate the rarity of short sequences in them. To capture this, we developed a metric of the rarity of each sequence in nature, the rarity index. We find that the frequency of certain dipeptides in rare oligopeptide sequences is hundreds of times lower than expected, which is not the case for any dinucleotides. We also generate predictive regression models that infer the rarity of nucleic and proteomic sequences across nature or within each domain of life and viruses separately. When examining each of the three domains of life and viruses separately, the *R*² performance of the model predicting rarity for 5-mer peptides from mono- and dipeptides ranged between 0.814 and 0.932. A separate model predicting rarity for 10-mer oligonucleotides from mono- and dinucleotides achieved *R*² performance between 0.408 and 0.606. Our results indicate that the mono- and dinucleotide composition of nucleic sequences and the mono- and dipeptide composition of peptide sequences can explain a significant proportion of the variance in their frequencies in nature.

## Introduction

Genomic and proteomic information is often measured using *k*-mers, which are contiguous sequences of length *k* composed of nucleotides in genomics or amino acids in proteomics. *K*-mers are distributed inhomogeneously in DNA and protein molecules (1–3). The frequency spectrum of *k*-mers can be analyzed, which displays the distribution of *k*-mer frequencies in a single genome or proteome (4–6). At the genomic level, important differences in dinucleotide abundance have been previously reported (7), while in mammalian genomes the GC and CpG contents have been shown to influence the frequency of *k*-mer sequences (8). Synonymous codon usage bias can also shape organismal genomes, as previously shown using the codon adaptation index, finding a significant impact on the genome composition (9,10). Other features, such as *cis*-regulatory elements, constrained genomic regions and pathogenic variant sites, also display specific *k*-mer preferences and shape the *k*-mer spectrum (11–16). At the proteomic level, the energy expenditure associated with each amino acid influences the *k*-mer peptide frequency spectrum (17). In addition, specific functional sites and common protein motifs account for overrepresented *k*-mers (18).

There are also *k*-mer sequences that are rare or absent from one or more genomes or proteomes. *K*-mer sequences that are absent from a genome or proteome are referred to as nullomers and nullpeptides, respectively (19–23). The absence of *k*-mer sequences has been previously attributed to selection constraints and hypermutation in nucleic sequences and to structural and chemical constraints in peptide sequences (24–26). *K*-mers absent from every genome or proteome are referred to as primes (19,25). Additionally, the distribution of peptide *k*-mers was previously studied across organismal proteomes, in which study quasi-primes were proposed as the shortest *k*-mer sequences, which are unique to a species’s genome or proteome and absent from every other organism (27–29). As an extension, certain *k*-mer sequences can be preferentially found within specific phylogenetic groups and a subset of *k*-mers are taxonomy specific (27). Previous work has also utilized *k*-mers for alignment-free comparison of *k*-mer contents between genomes or proteomes of different organisms (30–33).

**Figure 1.**
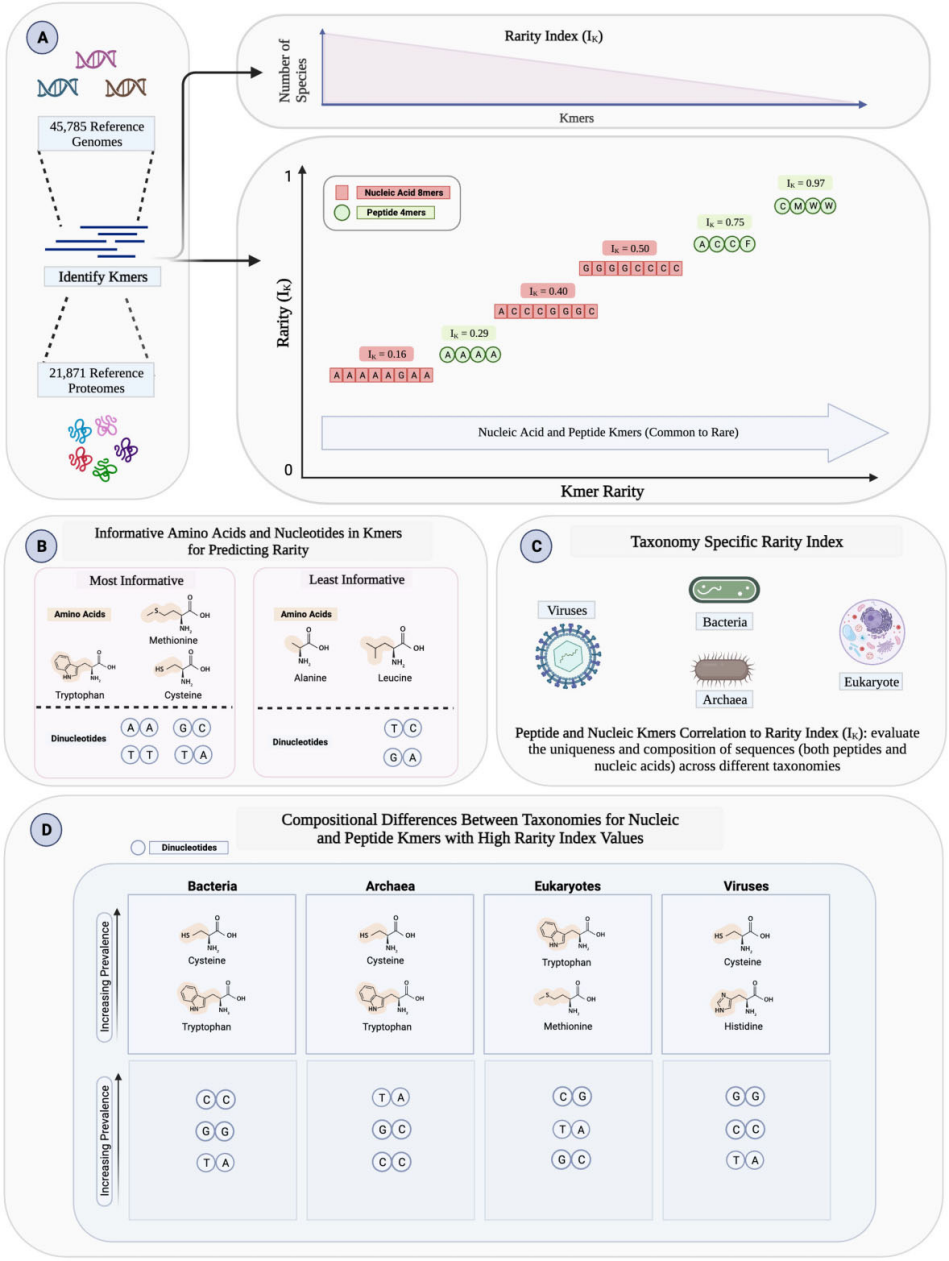
Schematic illustration of the rarity index, an estimate of the rarity of each *k*-mer peptide or nucleic sequence in nature. (**A**) Schematic displaying the identification of rarity index to depict the rarity of various peptide and nucleic acid *k*-mers. All *k*-mers were identified from 45 785 reference genomes and 21 871 reference proteomes. Nucleic acid and peptide *k*-mer rarity index values were identified on a scale from 0 to 1, with values closer to 1 indicating increased sequence absence across organisms. (**B**) Schematic showing the various amino acids and nucleotides in *k*-mers that predict rarity, specifying the most and least informative amino acids and dinucleotides. (**C**) Taxonomy-specific peptide and nucleic *k*-mers were determined to evaluate the uniqueness of sequences across various organismal groups. (**D**) Compositional differences across taxonomies were depicted to show that the decreasing prevalence of *k*-mers produces an increasing prevalence of these associated dinucleotides and peptides.

Here we introduce the rarity index, a measure of anti-popularity of genomic or proteomic sequences in nature (Figure 1). To calculate the rarity of *k*-mer sequences, we examine 45 785 reference genomes and 21 871 reference proteomes, spanning archaea, bacteria, viruses and eukaryotes, from which we extract the set of *k*-mers in each of them, to estimate the rarity of individual *k*-mers, within and across taxonomies. We find that there are significant compositional biases in rare sequences, in both peptide and nucleic *k*-mers. We generate predictive models that estimate the rarity of each *k*-mer sequence, trained on the primary mono- and dinucleotide contents of nucleic *k*-mers or using the amino acid and dipeptide contents of each peptide *k*-mer. Additionally, we generate models trained on physicochemical information of each peptide *k*-mer that achieve comparable results. These findings provide evidence that the rarity of biological information in nature can be inferred.

## Materials and methods

### Reference genomes and proteomes used

Collection of reference genomes was performed for the GenBank and RefSeq databases (34,35) as well as 104 reference genomes from the UCSC Genome Browser website. The GenBank database included 26 854 bacterial, 438 archaeal, 41 986 viral, 164 fungal, 5 plant and 1 invertebrate complete genomes. The RefSeq database included 23 120 bacterial, 388 archaeal, 11 128 viral, 19 fungal, 3 plant and 1 invertebrate complete genomes. Reference proteomes were downloaded from UniProt (https://ftp.uniprot.org/pub/databases/uniprot/current_release/knowledgebase/reference_proteomes/Reference_Proteomes_2022_03.tar.gz, release 2022_03, 19 September 2022) and included a total of 344 archaeal, 8623 bacterial, 2119 eukaryotic and 10 789 viral reference proteomes. The total number of reference proteomes examined totaled 21 875 species (36).

### Derivation of the *k*-mer profile of each genome and proteome

Identification of *k*-mers was performed as previously described in (27) for each genome and each proteome. For genomes, *k*-mer lengths between 6 and 12 bp were used. For proteomes, *k*-mer lengths of two to six amino acids were used.

### Derivation of the rarity index

Mathematically, the rarity index is defined below.

Let us define the set of all *k*-mers of length *k* present in a genome or proteome, represented by ${{S}^k}_{}$. Furthermore, we define $T = \{ {S_1^k,S_2^k, S_3^k, \ldots , S_n^k} \}$ as the set containing the sets of *k*-mers present in each genome or proteome in our reference database consisting of $n$ entries. The rarity index ${{I}_K}$ of a *k*-mer $K$ is defined as


(1) \begin{equation*}{{I}_{K }} = \frac{{\left| {1 \le i \le n:\ K \notin S_i^k} \right|}}{n}.\end{equation*}

The rarity index ${{I}_K}$ ranges between 0 and 1, with 0 indicating a sequence found in every organism and 1 indicating a sequence absent across every species. The concept applies to different categories of biological sequences, including *k*-mer peptides and nucleic acids.

### Calculation of the rarity index

The number of species each *k*-mer was found in was calculated. Based on this, the proportion of species a *k*-mer was not found in was calculated, from which we derived the rarity index (see Equation 1). Similarly, the rarity index was also calculated within available species of individual taxonomies, namely viruses, bacteria, eukaryotes and archaea. The frequency of each amino acid and each dinucleotide was calculated as a function of the rarity index in proteomes and genomes, respectively. We mapped each *k*-mer found in each species to its corresponding rarity index value. We estimated the total number of *k*-mers found in each species for each rarity index value.

To visualize the distribution across species, we defined rare *k*-mers as the top quartile of *k*-mers with the highest rarity index. We grouped rare *k*-mers to the third decimal digit of their rarity index, except for 12-mer oligonucleotides and 6-mer oligopeptides, which due to the higher overall rarity index we grouped to the fourth and fifth decimal digits, respectively. For each bin generated, we identified the number of occurrences of *k*-mers in that bin in each genome or proteome. To account for variability in genome or proteome size, the number of occurrences was divided by the total number of unique *k*-mers present in that genome or proteome. We then performed hierarchical clustering of the species using the ‘clustermap’ function of the Seaborn Python package (Figure 3A and D) (37).

### Calculation of physicochemical properties of peptides

For each peptide, the following biochemical properties were calculated using the Peptides package (38): the Boman index, the aliphatic index, the instability index, its hydrophobicity, the hydrophobic moment, the isoelectric point and its molecular weight.

### Oligopeptide and oligonucleotide regression models

The oligopeptide sequence-based predictive model used as feature space the amino acid and dipeptide contents of each oligopeptide, whereas the oligonucleotide sequence-based predictive model used as feature space the mono- and dinucleotide contents of each *k*-mer. The oligopeptide biochemical-based predictive model used as feature space the physicochemical properties. Ridge regression (L2 regularization) with strength regularization of *α* = 0.01 was used from the Scikit-learn package (39) using either the sequence-based features or the biochemical-based features. For each of the regression models, the coefficient scores of each feature were estimated, from which the most predictive features were identified. Random forest regression was implemented to capture nonlinear patterns for the biochemical-based model and for the sequence-based model using the Scikit-learn package (39). Regression models were trained and tested either within an individual taxonomy or across taxonomies. For determining feature importance, the instance attribute feature_importances_ was used. Cross-validation was performed for all models. For the models based on oligonucleotide sequence content, the mean absolute error was calculated with the mean_absolute_error function from the Scikit-learn package (39).

## Results

A *k*-mer sequence can be present in a set of genomes or proteomes, and its presence can reflect phylogenetic relationships and evolutionary history, biological mechanisms and functions or can be stochastic. Here, we define the rarity index as a measure of the rarity of each *k*-mer sequence across the examined organisms. The rarity index captures the fraction of genomes/proteomes in which a *k*-mer is not observed.

### Frequency patterns of *k*-mers across species

Using the set of 21 871 reference proteomes, we estimated the number of species each *k*-mer peptide was found in for peptide *k*-mer lengths between two and six amino acids. The *k*-mer length limit of six amino acids was used because at longer *k*-mer lengths most peptides are absent from most proteomes (Supplementary Figure S1). We also investigated 45 785 reference genomes and estimated the frequency of each nucleic *k*-mer across the examined species for *k*-mer lengths between 6 and 12 bp. The upper *k*-mer length limit of 12 bp was chosen to improve species differentiation, while taking into account computational constraints. This is because shorter *k*-mer lengths often result in the majority of genomes containing most of the possible *k*-mers, making it challenging to distinguish between species effectively (Supplementary Figure S2). We observe that the number of *k*-mers detected per species is highest across eukaryotes and lowest across viruses across the *k*-mer lengths studied when examining proteomes (Supplementary Figure S1) and genomes (Supplementary Figure S2). For instance, in 6-mer peptides there are on average 5 090 888, 1 070 044, 581 726 and 12 026 *k*-mers in eukaryotic, bacterial, archaeal and viral proteomes, respectively (Supplementary Figure S1). Furthermore, in 12-mer nucleic *k*-mers there are 14 248 979, 4 444 638, 3 225 470 and 83 603 different *k*-mers in eukaryotic, bacterial, archaeal and viral genomes (Supplementary Figure S2). This is an expected result, stemming from differences in genome and proteome sizes between species in different taxonomic groups.

**Figure 2.**
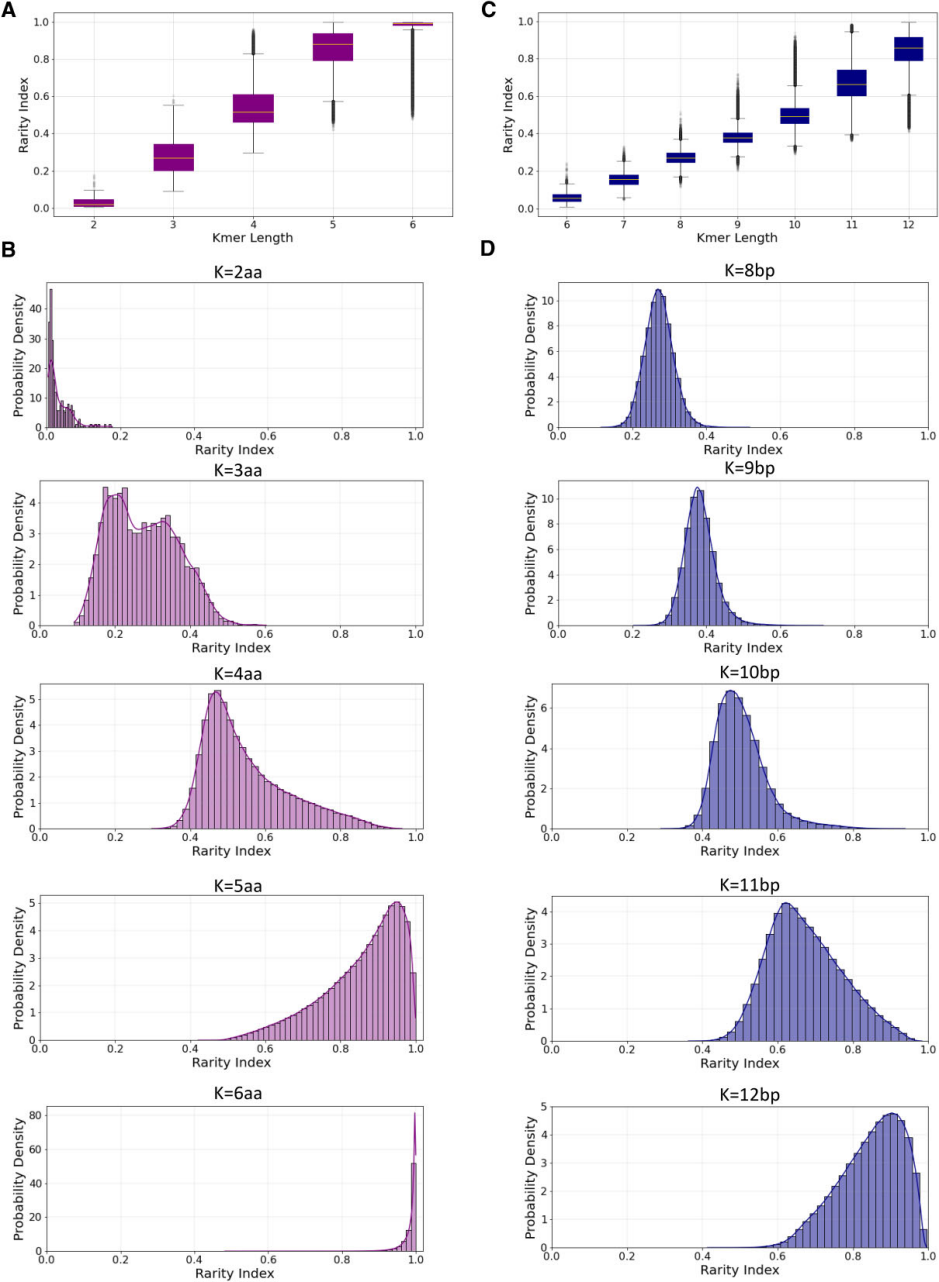
Oligopeptide and oligonucleotide rarity *k*-mer profiles. (**A**) The rarity index as a function of peptide *k*-mer length. (**B**) Probability density for the rarity *k*-mer profile for oligopeptide lengths of two to seven amino acids. (**C**) The rarity index as a function of nucleic *k*-mer length. (**D**) Probability density for the rarity *k*-mer profile for oligonucleotide lengths of 6–12 bp. A rarity index of 0 indicates that a sequence is found in every species and a rarity index of 1 indicates that a sequence is absent across all species.

Next, for each *k*-mer peptide we estimated its rarity index (${{I}_K}$), as a measure of the rarity of individual *k*-mer peptide sequences in nature. We observe that the rarity index of the *k*-mer peptides increased as a function of *k*-mer length and shifted from 0 to 1 (Pearson correlation = 0.991, *P* < 0.0001; Figure 2A and B and Supplementary Figure S3A). We also observe that after the *k*-mer length of four amino acids, the majority of *k*-mers are absent from most proteomes, while the first peptide prime sequences, which are absent from every proteome, appear at the *k*-mer length of six amino acids, having a rarity index of 1. Also, the likelihood of encountering a particular *k*-mer sequence decreases with increased *k*-mer length, because the *k*-mer space increases with an exponential pattern, which accounts for the shift in the rarity index for longer *k*-mer lengths.

From the frequency of each *k*-mer across the reference genomes, we derived the rarity index score of each nucleic *k*-mer. Similarly to the proteome analysis, we find that the number of species each *k*-mer appears in decreases as a function of *k*-mer length, and the rarity index distribution converges toward 1 (Pearson correlation = 0.992, *P* < 1.3e–05; Figure 2C and D and Supplementary Figure S3B). These results also indicate that, with the exception of eukaryotes, after 10 bp length, most potential *k*-mers are absent from a genome and that longer *k*-mers have a higher rarity index across sequence types. Additionally, a small subset of nucleic and peptide *k*-mers remains highly frequent, even at longer *k*-mer lengths, resulting in a negative skew with a long tail distribution (Figure 2B and D), which likely reflects repetitive or biologically functional *k*-mer sequences.

### Rare peptide and nucleic sequences have a distinct sequence composition

**Figure 3.**
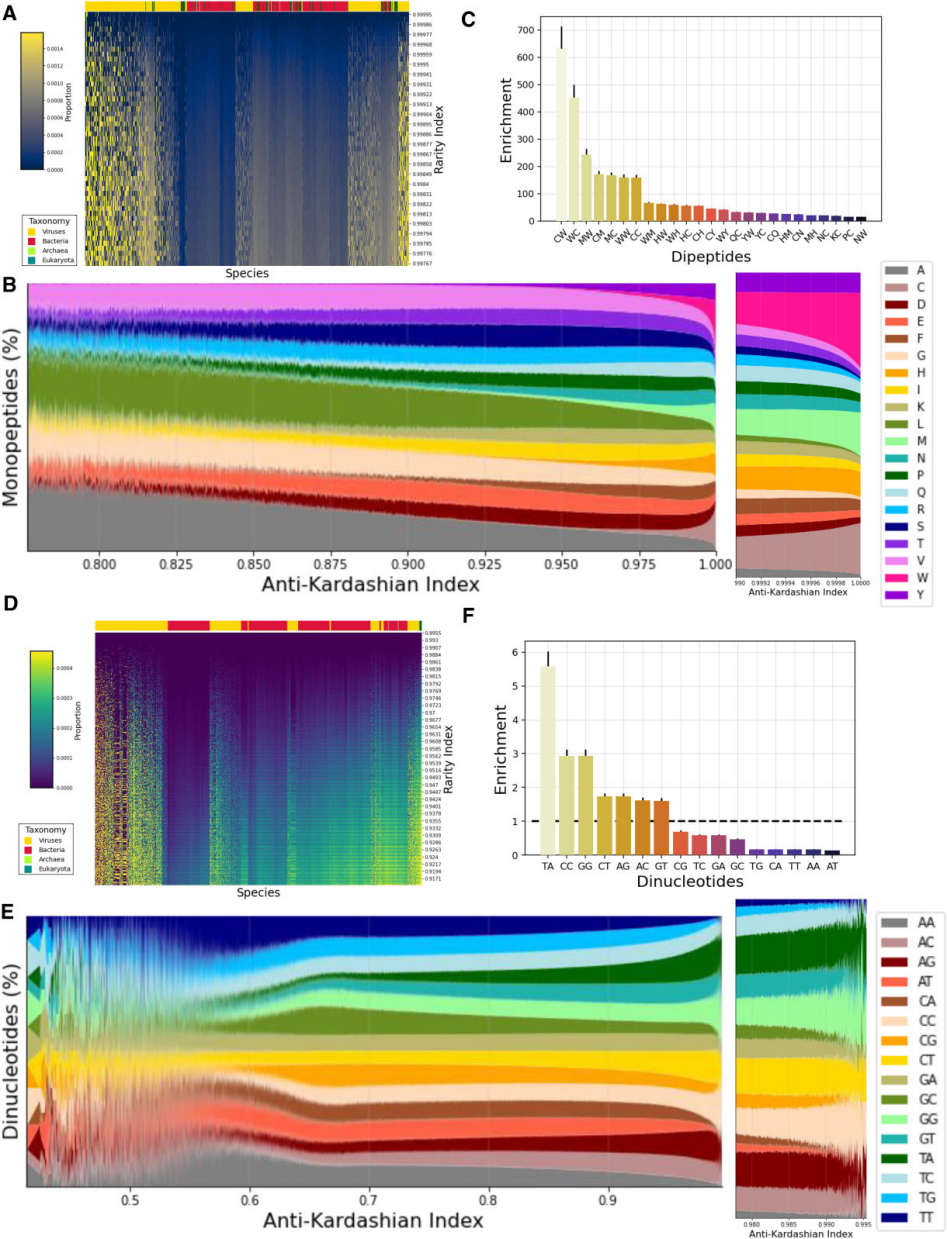
The rarity index scores across *k*-mers in proteomes and genomes and across the taxonomic subgroups. (**A**) Occurrences of top quartile of 6-mer oligopeptides with the highest rarity index score detected in each species. Number of occurrences is normalized by the total number of unique *k*-mers present in the corresponding proteome and indicated as proportion. Color bar indicates the taxonomic group, namely viruses, bacteria, archaea and eukaryotes. (**B**) Association between the rarity index and the amino acid and dipeptide contents of *k*-mers in proteomes and genomes. The frequency of each amino acid is estimated for different rarity index scores. Color bar indicates each amino acid. (**C**) Enrichment of dipeptides for frequencies in *k*-mer sequences with rarity index >0.95 over sequences with rarity index <0.9, for *k*-mer length of five amino acids. (**D**) Occurrences of top quartile of 12-mer oligonucleotide *k*-mers with the highest rarity index score detected in each species. Number of occurrences is normalized by the total number of unique *k*-mers present in the corresponding genome and indicated as proportion. Color bar indicates the taxonomic group, namely viruses, bacteria, archaea and eukaryotes. (**E**) The frequency of each dinucleotide is estimated for different rarity index scores. Color bar indicates each dinucleotide. (**F**) Enrichment of dinucleotides for frequencies in *k*-mer sequences with rarity index >0.99 over sequences with rarity index <0.5, for 12 bp *k*-mer length.

Furthermore, we examined the rarity index of peptide and nucleic *k*-mers across each species, spanning the three domains of life and in viruses. For peptide sequences, we find that clustering species based on the top quartile of oligopeptides with the highest rarity index score separates the taxonomies (Figure 3A and Supplementary Figure S4). We were also interested in investigating whether the rarity of *k*-mer sequences can be explained by their sequence composition. We therefore examined the amino acid and dinucleotide contents of peptide and nucleic *k*-mers as a function of the rarity index. We find that for peptides, tryptophan (W), methionine (M) and cysteine (C) increase in proportion for *k*-mers of higher rarity index, whereas alanine and leucine disappear at the *k*-mers with the highest rarity index across the *k*-mer lengths studied (Figure 3B and Supplementary Figure S5). In particular, above the 99th percentile of the rarity index, which reflects the rarest peptide sequences, tryptophan, methionine and cysteine increase precipitously and capture a significant proportion of the amino acid space. At dipeptides, the bias is even greater, with CW, WC and MW having 631-fold, 452-fold and 245-fold enrichment, respectively, in the frequency for *k*-mers with a rarity index >0.95 over sequences with a rarity index <0.9 (Figure 3C). These results indicate that rare or absent sequences are more likely to have specific amino acid or dipeptide content, which aligns with the known variations in the frequency of different amino acids.

When clustering based on the rarity index of the top quartile of oligonucleotide *k*-mers with the highest rarity index score across the organismal reference genomes, we also observe taxonomic clustering (Figure 3D and Supplementary Figure S6). We also investigate the frequencies of dinucleotides in nucleic *k*-mers as a function of their rarity index and observe significant differences across the continuum. Increased frequencies of TA and GG/CC *k*-mers are associated with higher rarity index (Figure 3E and Supplementary Figure S7). In contrast, AA/TT are enriched at *k*-mers with low rarity index, which is particularly evident above the 99th percentile across *k*-mer lengths (Figure 3E and Supplementary Figure S7). For instance, for TA and CC/GG we observe 5.58-fold and 2.93-fold enrichment for 12-bp *k*-mers with a rarity index >0.99 over sequences with a rarity index <0.5 (Figure 3F). These results indicate differences of up to 5.58-fold in the nucleotide composition of rare nucleic *k*-mers. The findings from peptide *k*-mers and nucleic *k*-mers are fundamentally distinct and should not be compared directly.

### Comparison of *k*-mer frequency patterns between species of different taxonomies

**Figure 4.**
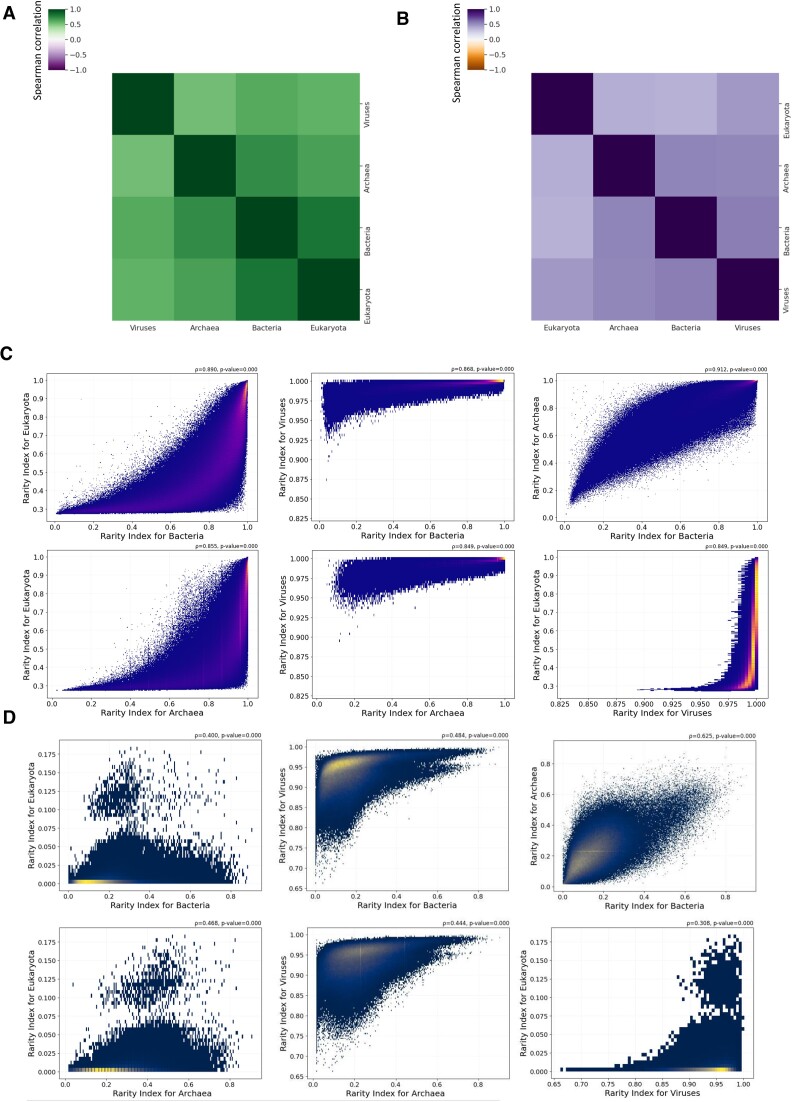
The rarity index between taxonomic subgroups for peptide and nucleic *k*-mers. (**A**) Spearman correlation matrix of the rarity index between viruses, bacteria, archaea and eukaryotes for peptide *k*-mers. Results shown for six-amino-acid *k*-mers. (**B**) Spearman correlation matrix of the rarity index between viruses, bacteria, archaea and eukaryotes for 12-mer nucleic *k*-mers. (**C**) Scatter plot showing the correlation of the rarity index between pairs of taxonomic subgroups for peptide *k*-mers. Coloring was performed according to the density of data from blue (low density) to yellow (high density). Results shown for 10-bp *k*-mers. (**D**) Scatter plot showing the correlation of the rarity index between pairs of taxonomic subgroups for nucleic *k*-mers. Coloring was performed according to the density of data from blue (low density) to yellow (high density). Results shown for five-amino-acid *k*-mers. (C, D) Spearman correlation and associated *P*-values are shown.

We next estimated the rarity index of peptide and nucleic *k*-mers within individual taxonomies, namely archaea, bacteria, eukaryotes and viruses, and examined the correlation of the taxonomic rarity index between them. Overall, we observe significant positive correlations between all the taxonomies for both peptide and nucleic *k*-mers (Figure 4A and B). Specifically, for both peptide and nucleic *k*-mers, the comparison of the rarity index between taxonomic groups revealed positive correlations between all taxonomies, with the viruses displaying the weakest correlations with the other taxonomic groups (Figure 4A and B). The strongest correlations were observed between archaea and bacteria (Figure 4A and B), which could be attributable to the more similar genome and proteome lengths. We also observe significant deviations for specific *k*-mers for their taxonomic rarity index, when performing pairwise comparisons between the three domains of life and viruses (Figure 4C and D), which could reflect compositional differences between the taxonomies but also functional differences of biological importance.

We were also interested in investigating taxonomic differences in the amino acid and dinucleotide frequencies across the rarity spectrum, which could explain the differences in *k*-mer rarity between the taxonomies. We therefore examined the amino acid and dinucleotide contents of *k*-mers within individual taxonomies as a function of the rarity index scores. For amino acids, we find that in viruses, alanine and glycine are the primary amino acids found in commonly used *k*-mers, whereas other amino acids are less likely to be shared between viral proteomes (Supplementary Figure S8). Bacteria, archaea and eukaryotes show similar profiles, with tryptophan, methionine and cysteine being overrepresented in *k*-mers with the highest rarity index scores (Supplementary Figure S8). In contrast, peptide *k*-mers with high rarity index scores in viruses do not show a preference for tryptophan, methionine or cysteine (Supplementary Figure S8). These compositional differences for rare *k*-mers between viruses and other taxonomic groups account for the weaker correlation of the taxonomic rarity index in viruses with the other taxonomies (Figure 4A).

Next, we examined nucleic *k*-mers for dinucleotide usage differences between taxonomies. We find that multiple dinucleotides show differences in their frequency profiles, including GC/CG, which are overrepresented in eukaryotes relative to bacteria for high rarity index (Supplementary Figure S9). In contrast, bacteria, archaea and viruses show higher levels of GG/CC at high rarity index scores relative to eukaryotes (Supplementary Figure S9). We therefore note significant compositional differences between taxonomies for nucleic *k*-mers with high rarity index.

### Sequence-based and physicochemical-based models of peptide *k*-mer rarity in nature

Next, we investigated whether we can predict the rarity of each peptide *k*-mer in nature using as features its mono- and dipeptide sequence profiles. Using ridge regression, we generated a model that predicts the rarity of peptide *k*-mers, as measured by the rarity index, for peptide *k*-mer lengths of three to six amino acids. The coefficient of determination (*R*²) of the model ranged between 0.972 and 0.561 for peptide *k*-mer lengths of three to six amino acids, respectively (Supplementary Figure S10), indicating that the amino acid and dipeptide profile is largely predictive of the rarity of a peptide sequence in nature. We also examined the most informative coefficients of the ridge regression model. We observe that the most informative features are amino acids relative to dipeptides, including cysteine, tryptophan, alanine and leucine, of which alanine and leucine are associated with a lower rarity index, whereas cysteine and tryptophan are associated with a larger rarity index (Supplementary Figure S11A and B). These results are consistent with our earlier findings regarding differences in the amino acid composition of rare peptide *k*-mers.

**Figure 5.**
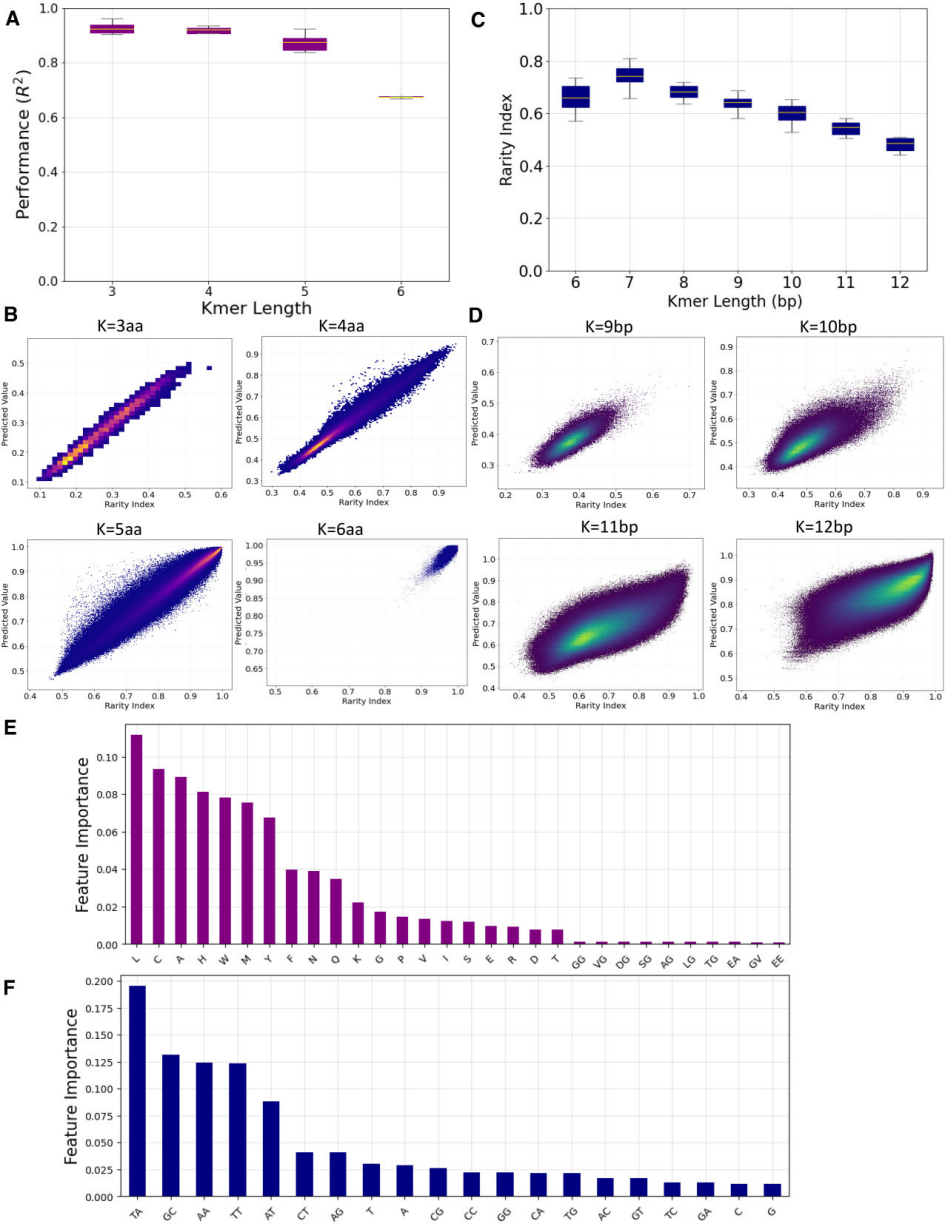
Predictive regression model determines the rarity of oligonucleotide sequences in nature. (**A**) Regression model for the rarity of 6-mer peptides. (**B**) Regression model for the rarity of 10-mer oligonucleotides. In panel (B), coloring was performed according to the density of data from blue (low density) to yellow (high density). (**C**, **D)** Coefficients of regression model for oligopeptides and oligonucleotides. In panel (D), coloring was performed according to the density of data from blue (low density) to yellow (high density). (**E**) Feature importance for the random forest regression model predicting the rarity of six-amino-acid peptides. (**F**) Feature importance for the random forest regression model predicting the rarity of 12-bp nucleic *k*-mers.

Next, in order to capture nonlinear relationships, we developed a random forest regression model. We find that the model’s performance measured by *R*² ranged between 0.968 and 0.816 for *k*-mer lengths of three and six amino acids (Figure 5A and B). When examining the feature importance in the model, we find amino acids to be more important in the predictive model than dipeptides across *k*-mer lengths (Figure 5E and Supplementary Figure S12). Therefore, we conclude that the rarity of peptide *k*-mers can be largely predicted using the amino acid and dipeptide contents as features, with the amino acid composition of a peptide *k*-mer providing the most significant predictive values.

We examined whether the physicochemical properties of the constituent amino acids of each peptide *k*-mer can predict the rarity index score of each peptide *k*-mer. The physicochemical features that were calculated for each *k*-mer included the molecular weight, the isoelectric point, the charge, the hydrophobicity, the hydrophobic moment, the Boman index, the aliphatic index and the instability index. We next generated predictive models, based on these physicochemical properties of each oligopeptide. We report that the performance of a ridge regression model (*R*²) ranged in 3-mers and 6-mers between 0.426 and 0.200 (Supplementary Figure S13A and B). We observe that in contrast to the sequence-based model, a ridge regression has limited predictive power when using the set of physicochemical features across the examined *k*-mer lengths. Therefore, we also developed a random forest regression model that can capture nonlinear relationships. We find that the random forest regression model trained on the physicochemical features achieves comparable results to the sequence-based model, with *R*² ranging between 0.950 and 0.788 for *k*-mer lengths of three and six amino acids (Supplementary Figure S13C and D). We also note that physicochemical properties of peptides are linked to their amino acid profile and are correlated features. We conclude that sequence-based and physicochemical-based regression models are predictive of peptide *k*-mer rarity in nature and capture most of the variance between organismal proteomes.

### Sequence-based models of nucleic *k*-mer rarity in nature

We were interested in examining whether there are sequence-based features that can be useful in predicting the rarity of nucleic sequences. To that end, we developed a sequence-based model that can predict the rarity of oligonucleotide *k*-mer sequences across the 45 975 reference genomes studied. As features, we used the mono- and dinucleotide profiles for each oligonucleotide *k*-mer sequence of length between 6 and 12 bp. We first implemented a ridge regression model, to capture linear relationships, which was trained and tested separately for each *k*-mer length. The model achieved *R*² performance between 0.331 and 0.561 for *k*-mer lengths of 6 and 12 bp, respectively (Supplementary Figure S10C and D). As a result, we conclude that a linear regression model based on mono- and dinucleotide features cannot accurately predict the rarity of nucleic *k*-mer sequences across organismal genomes.

To capture nonlinear relationships, we used a random forest regression model, which significantly improved the performance of our model, across the *k*-mer lengths studied. In particular, the model achieved *R*² between 0.67 and 0.48 for *k*-mer lengths of 6 and 12 bp, outperforming the ridge regression model by up to 55% (Figure 5C and D). These findings indicate that the proportion of species in which nucleic sequences between 6 and 12 bp are found can be inferred to a significant extent from the mono- and dinucleotide composition of these sequences.

Importantly, we examined the relative importance of each mononucleotide and dinucleotide in the performance of the random forest regression model. We report that the dinucleotides TA, GC and TT/AA are the most informative features in predicting the rarity of 12-mer sequences in our model (Figure 5F and Supplementary Figures S11 and S14). In contrast, G and C mononucleotides and GA/TC dinucleotides are the least informative features in our models (Figure 5F and Supplementary Figures S11 and S14). These results could reflect the significance of the rarity of the TA dinucleotide across most organismal genomes, which has been previously reported (40), and the influence of repetitive sequences, which can be captured in repetitions of TT/AA motifs.

**Figure 6.**
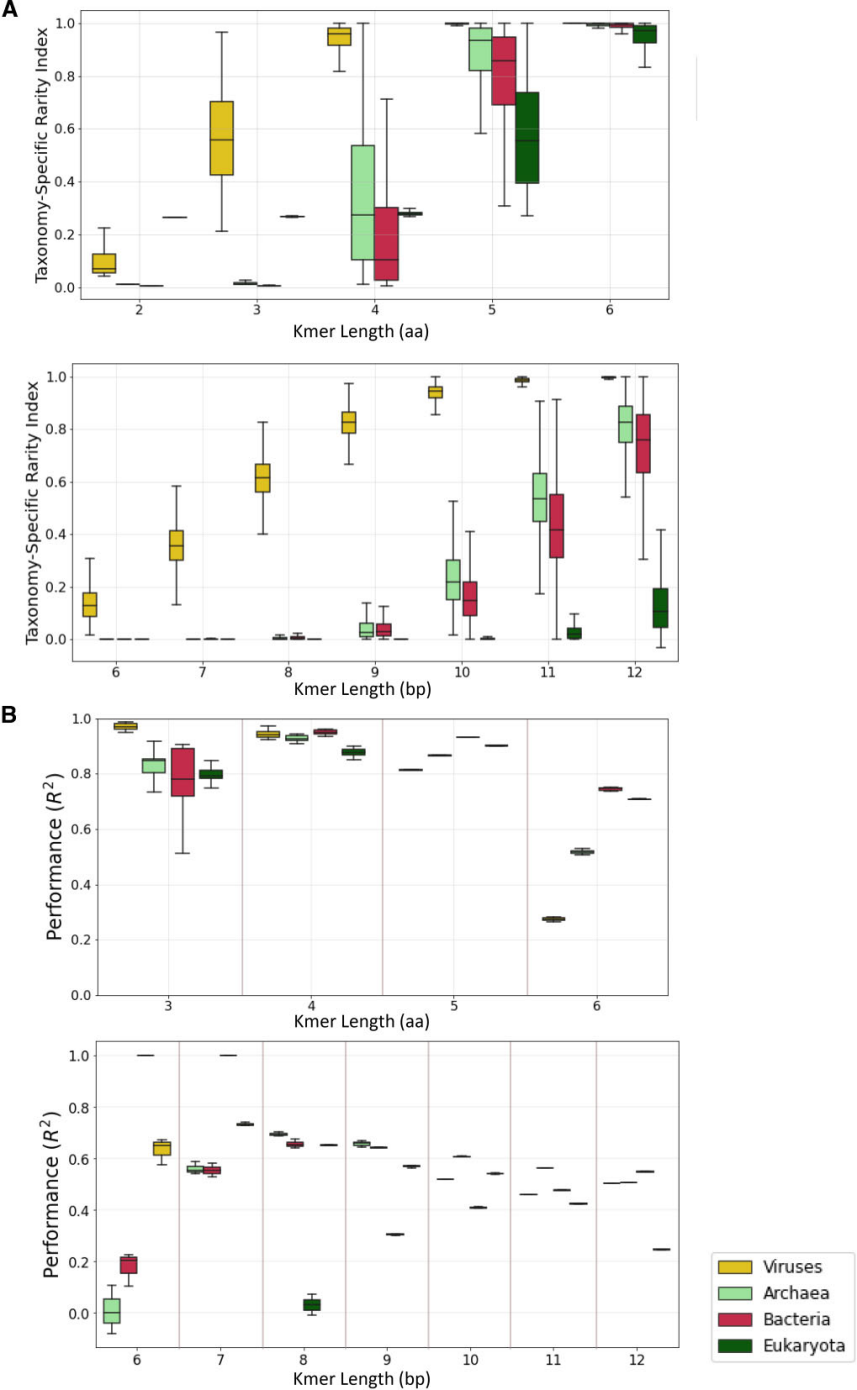
Rarity index in taxonomic subgroups. (**A**) The rarity index as a function of peptide and nucleic *k*-mer length for individual taxonomies. (**B**) Predictive random forest regression models for the taxonomy-specific rarity of peptide *k*-mers and nucleic *k*-mers, across organismal genomes.

To further analyze differences between taxonomies, we calculated the peptide and nucleic rarity indexes within individual taxonomies, namely for eukaryotes, archaea, bacteria and viruses (Figure 6A). We also identified the set of peptide and nucleic *k*-mers that are rarest in one taxonomic group, between archaea, viruses, bacteria and eukaryotes, but remain common in the others (Supplementary Tables S1 and S2). Finally, we generated random forest classification models, to predict the rarity index, within individual taxonomies. We report that the *R*² achieved for 5-mer peptides based on their mono- and dinucleotide contents ranged between 0.814 and 0.932 (Figure 6B). For 10-mer oligonucleotides, the models based on mono- and dinucleotide contents achieved *R*² ranging between 0.408 and 0.606 in eukaryotes and viral taxonomic groups (Figure 6B). We note that in some cases we observe poor *R*² performance, for example in 6-mer oligonucleotides for archaea and bacteria, as well as 8-mer oligonucleotides for eukaryotes. Further investigation revealed that for these taxonomies at this length, the majority of *k*-mers have a taxonomic rarity index extremely close to 0. This results in a very small total variance and thus poor *R*² performance, even though the models’ predictions are very close to the actual values, as indicated by the mean absolute error, which is $2.08 \times {{10}^{ - 5}}$ for 6-mers in archaea, $5.01 \times {{10}^{ - 5}}$ in bacteria and $2.07 \times {{10}^{ - 5}}$ in 8-mer eukaryotes (Supplementary Figure S15). A similar pattern is observed for 12-mer oligonucleotides for viruses, for which most *k*-mers have a taxonomic rarity index of ∼1.

## Discussion

In this work, we have described the rarity index, a measure of the rarity of *k*-mers, including oligopeptides and oligonucleotides, across organisms in natural sequences. In total, we analyzed 45 785 reference genomes and 21 871 reference proteomes and developed predictive models that can infer the rarity of a given sequence across the examined species. For oligopeptides, the amino acid composition of a *k*-mer is the primary determinant of its rarity in nature, whereas for oligonucleotides we observe that its dinucleotide composition can partially account for its rarity. For peptide sequences, we also develop predictive models based on the biochemical properties of each oligopeptide sequence, and the performance of these models is close to those of the sequence-based models.

For peptide *k*-mers, differences in amino acid molecular weight, energy expenditure, acquisition or synthesis costs influence the oligopeptide frequency spectrum in each organism (17,41,42) and likely account for the high predictive power of our models. For nucleic *k*-mers, the *k*-mer frequency spectra show differing distributions between species, including bimodal and unimodal spectra (5), and could account for differences in the rarity index scores when comparing different taxonomies. Dinucleotide compositional heterogeneity has been previously described across multiple species. Findings include the rarity of the TA dinucleotide across most organismal genomes (40) and the rarity of CG dinucleotides in vertebrates (43), both of which are consistent with our findings.

Previous studies have indicated that *k*-mer distribution and frequency differences between species can reflect differences in biological processes that are operative, including horizontal gene transfer events (44), paralogous duplication events and selection (45), transposable element activity (46), environmental constraints and DNA repair differences (47), among others. Another important factor, the contribution of which needs to be examined in future work, is synonymous codon usage biases that have been previously shown to significantly impact genome composition and the proportion of the genomic space that is protein coding (9,10). *K*-mers are unevenly distributed in organismal genomes and proteomes, including in functional genomic subcompartments (11,16,45,48). Furthermore, characteristics of genomic elements such as the presence of intronic sequences or the length of untranslated regions are associated with the complexity of an organism (16,49–51), which in turn influences the frequency and type of *k*-mers observed in different species and taxonomies. Quantification of nucleic and peptide *k*-mer frequency biases, either within certain taxonomic groups or across them, can also reflect functional motifs as previously indicated (52–56). Therefore, future work is required to model the contribution of the plethora of biological factors, and their contribution, in the rarity of nucleic and peptide *k*-mers across nature.

The rarity index can provide insights into evolutionary relationships between species, as it enables examining the set of *k*-mers that are rare and only shared in a minority of organisms. Differences in genome and proteome sizes can influence *k*-mer profiles, and such differences increase between taxonomic subgroups (3,57,58) and therefore can also influence the rarity index and are addressed by the derivation of the rarity index at taxonomic subgroups. In future work, we would be interested in examining other finer taxonomic subdivisions, including potentially kingdoms and phyla. Indeed, we find significant differences between the three domains of life and viruses, which likely reflect differences in operative processes and selection constraints that shape their genomes and proteomes. Further work is also required to study the potential functions of rare and taxonomically biased *k*-mers and their potential functional effects within evolutionary lineages.

The rarity index could also have multiple practical applications, including in the development of biomarkers for the detection of pathogens and in wildlife conservation by selecting rare *k*-mers that are highly informative. Genetic engineering applications such as those using CRISPR could also benefit by selecting targets found in only a subset of the species. It will also be of interest to use the rarity index in metagenomic data and genome scaffolds. Currently, the rarity index also entails biases associated with the availability of reference genomes. Finally, as more reference genomes and proteomes become available, the rarity index can be updated to more thoroughly reflect the rarity of *k*-mers in nature.

In summary, our work provides an index that estimates the rarity of each *k*-mer in DNA and amino acid sequences and predictive models indicate that we can largely estimate the rarity of *k*-mer sequences.

## Supplementary Material

lqae029_Supplemental_File

## Data Availability

The code for this work can be found at https://github.com/Georgakopoulos-Soares-lab/RarityIndex and https://doi.org/10.5281/zenodo.10821875.
