# Supplementary material for: The determinants of the rarity of nucleic and peptide short sequences in nature
Source: NAR Genom Bioinform. 2024 Apr 4;6(2):lqae029. doi: 10.1093/nargab/lqae029 (PMC10993293; doi:10.1093/nargab/lqae029)
Supplement: lqae029_Supplemental_File [file lqae029_supplemental_file.docx]

# **Supplementary Material**

**
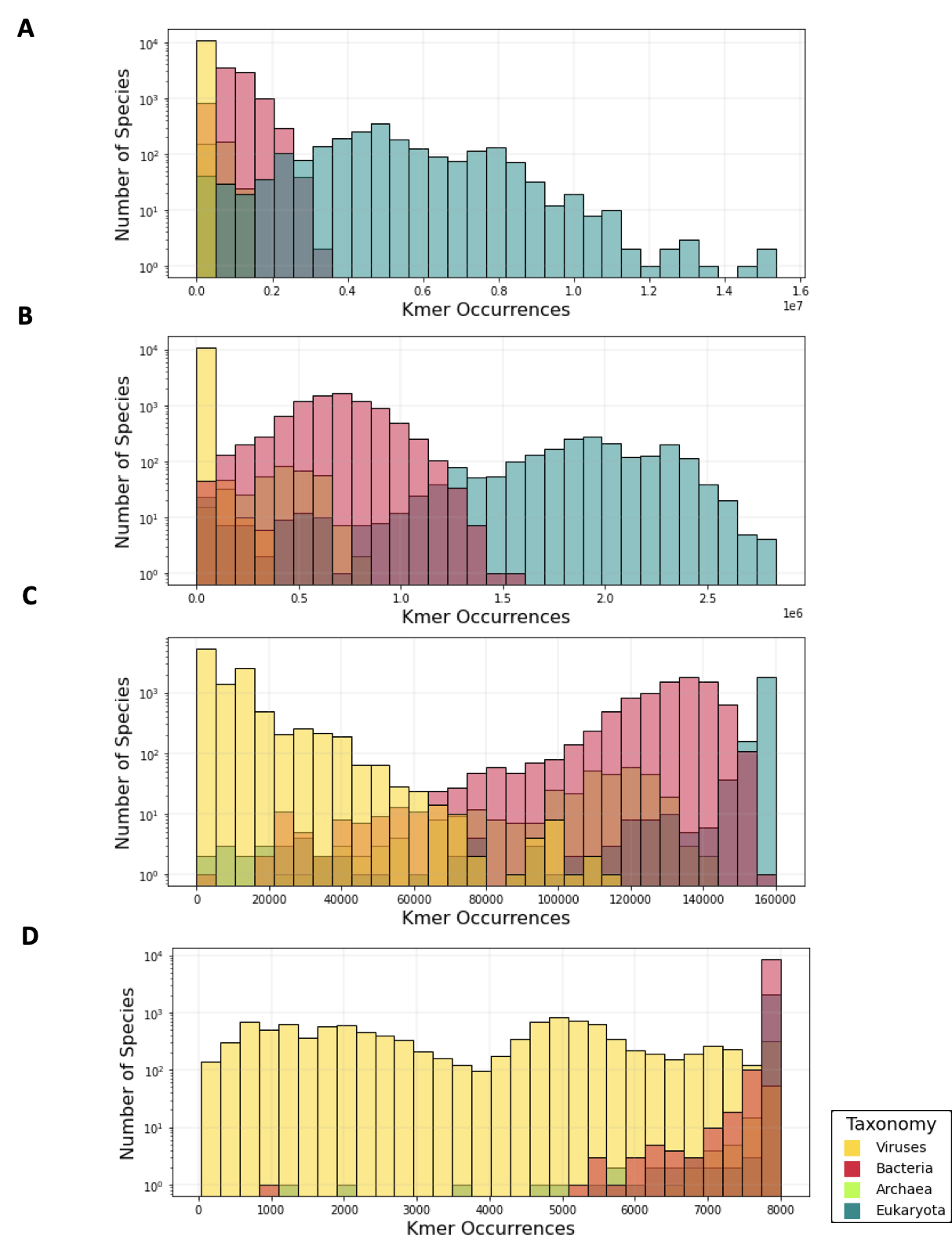
**

**Supplementary Figure 1: Number of species each peptide kmer was identified in.** Results shown for peptide kmer lengths of: **A.** six, **B.** five, **C.** four, **D.** three amino acids.


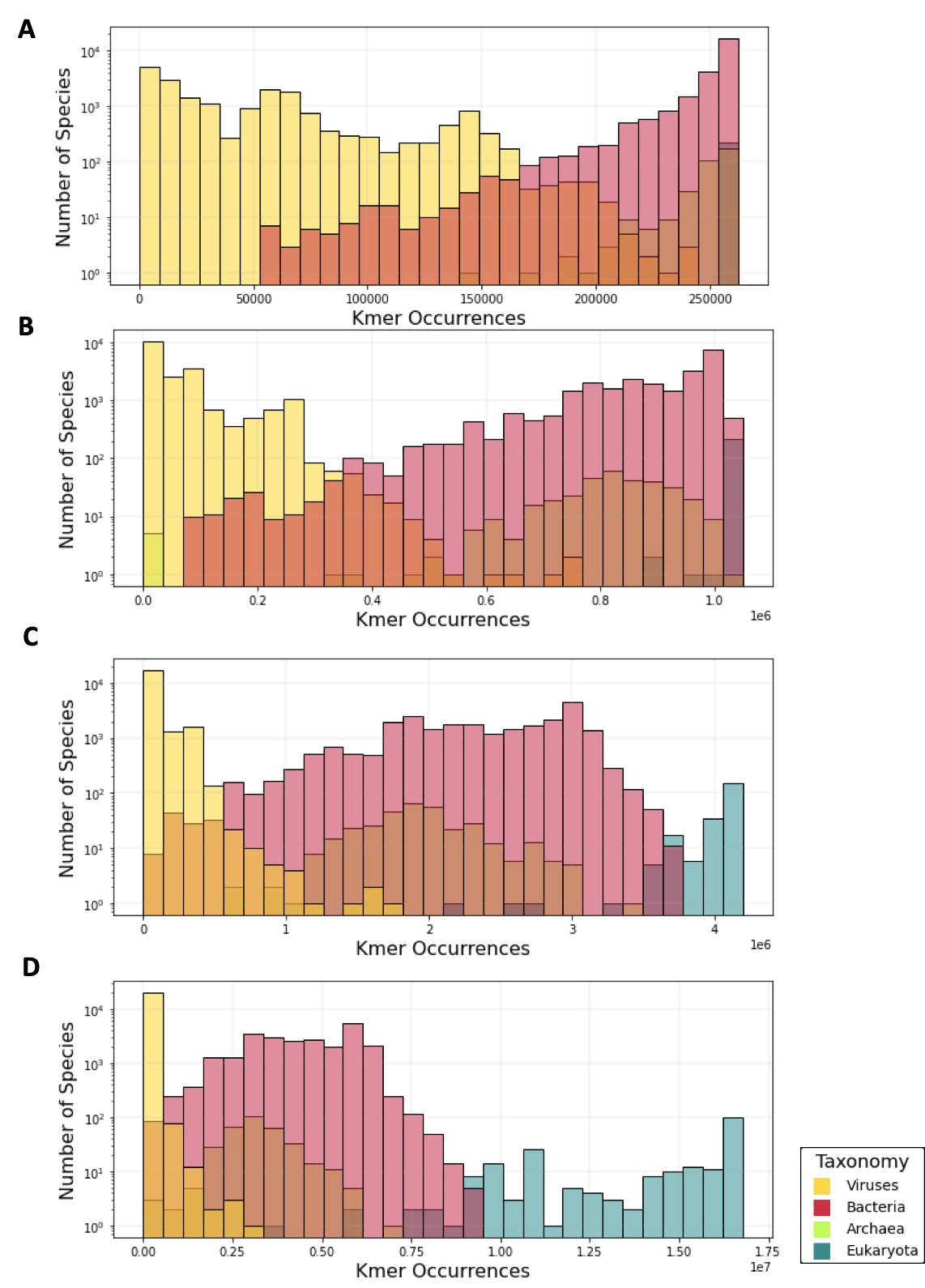


**Supplementary Figure 2: Number of species each nucleic kmer was identified in.** Results shown for nucleic kmer lengths of: **A.** nine, **B.** ten, **C.** eleven, **D.** twelve bps.

**A**

**
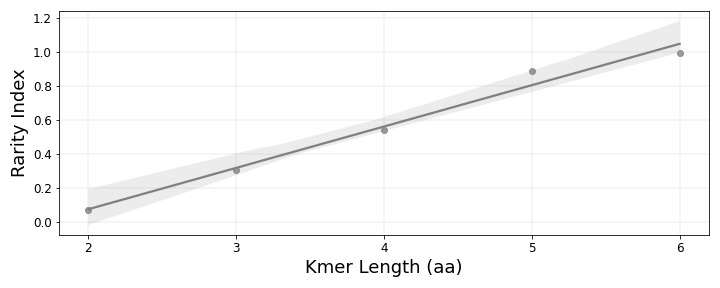
**

**B**

**
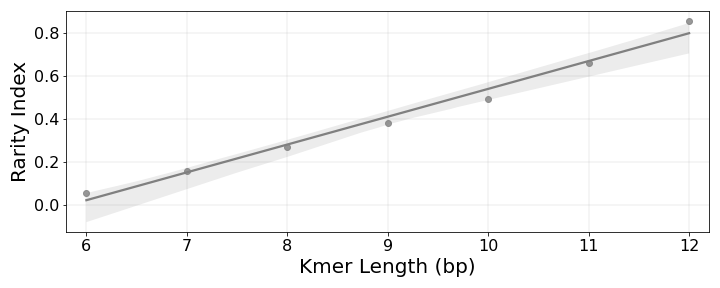
**

**Supplementary Figure 3: Association of the Rarity Index and kmer length for A. peptide kmers, B. nucleic kmers.** Error lines show 99 percentile confidence intervals.


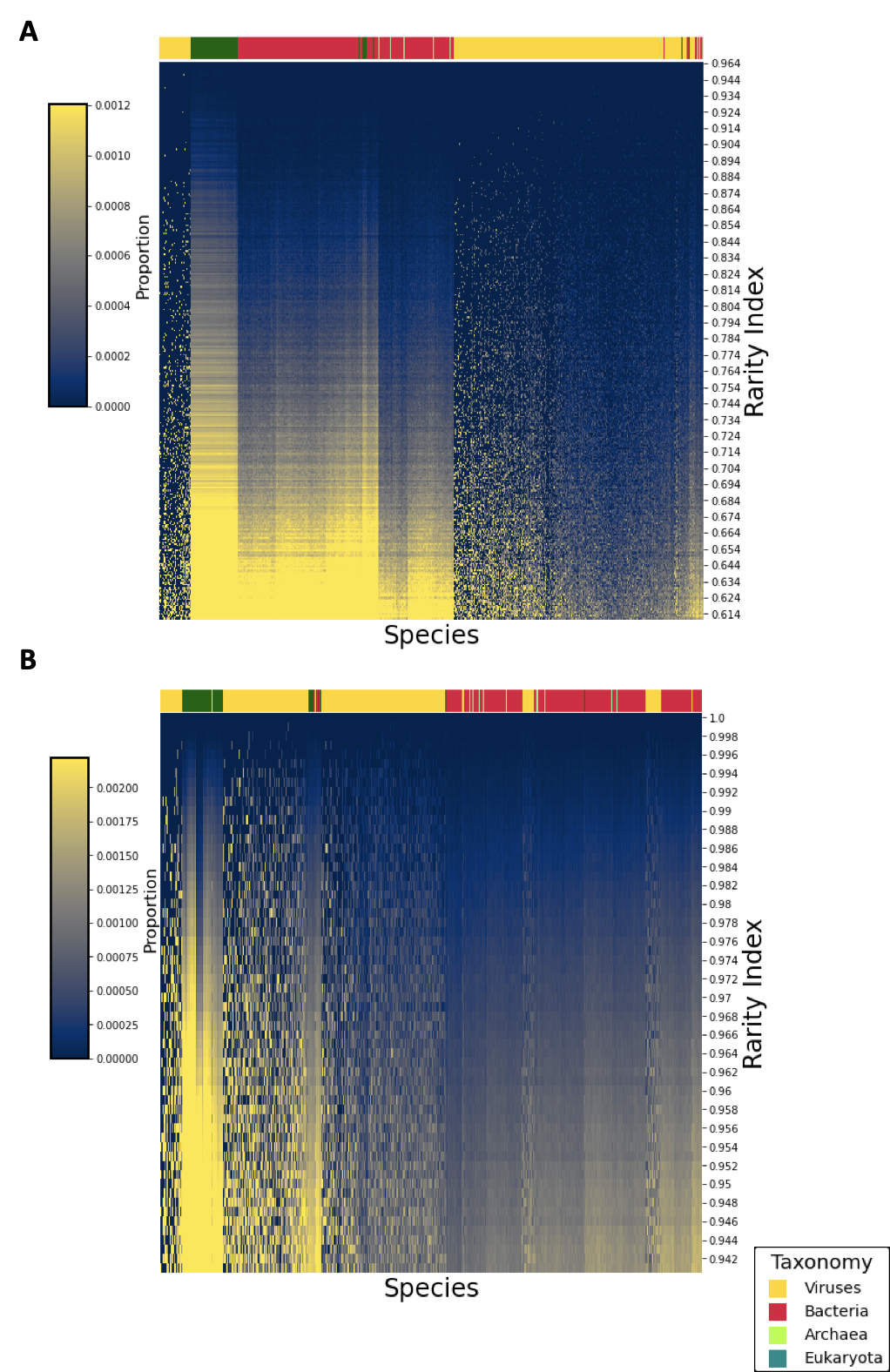


**Supplementary Figure 4: Occurrences of top quartile of oligopeptides with the highest Rarity index score detected in each species.** Number of occurrences is normalized by the total number of unique kmers present in the corresponding proteome and indicated as proportion. Results shown for: A. four, B. five amino acids peptide kmer length. Color bar indicates the taxonomic group, namely viruses, bacteria, archaea and eukaryotes.


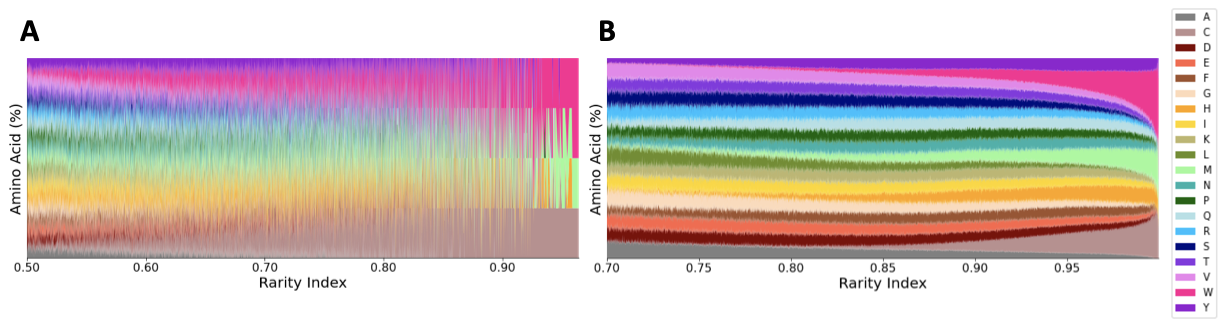


**Supplementary Figure 5: Association between the Rarity Index and amino acid content of kmers in proteomes.** Results shown for: **A.** 4 amino acids, **B.** 5 amino acids.


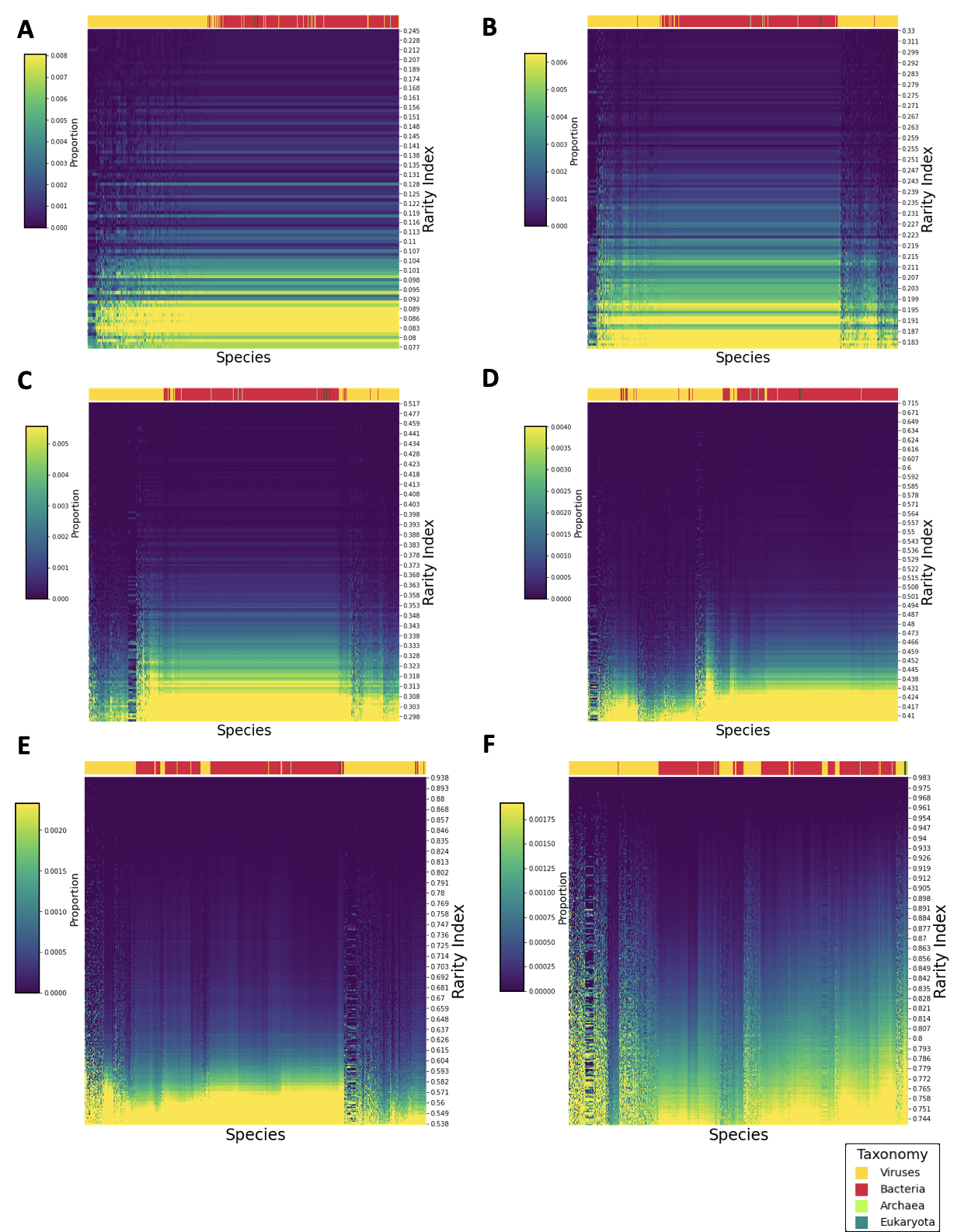


**Supplementary Figure 6: Occurrences of top quartile of oligonucleotides with the highest Rarity index score detected in each species.** Number of occurrences is normalized by the total number of unique kmers present in the corresponding genome and indicated as proportion. Results shown for: **A.** six, **B.** seven, **C.** eight, **D.** nine, **E.** ten, **F.** eleven bps kmer length. Color bar indicates the taxonomic group, namely viruses, bacteria, archaea and eukaryotes.


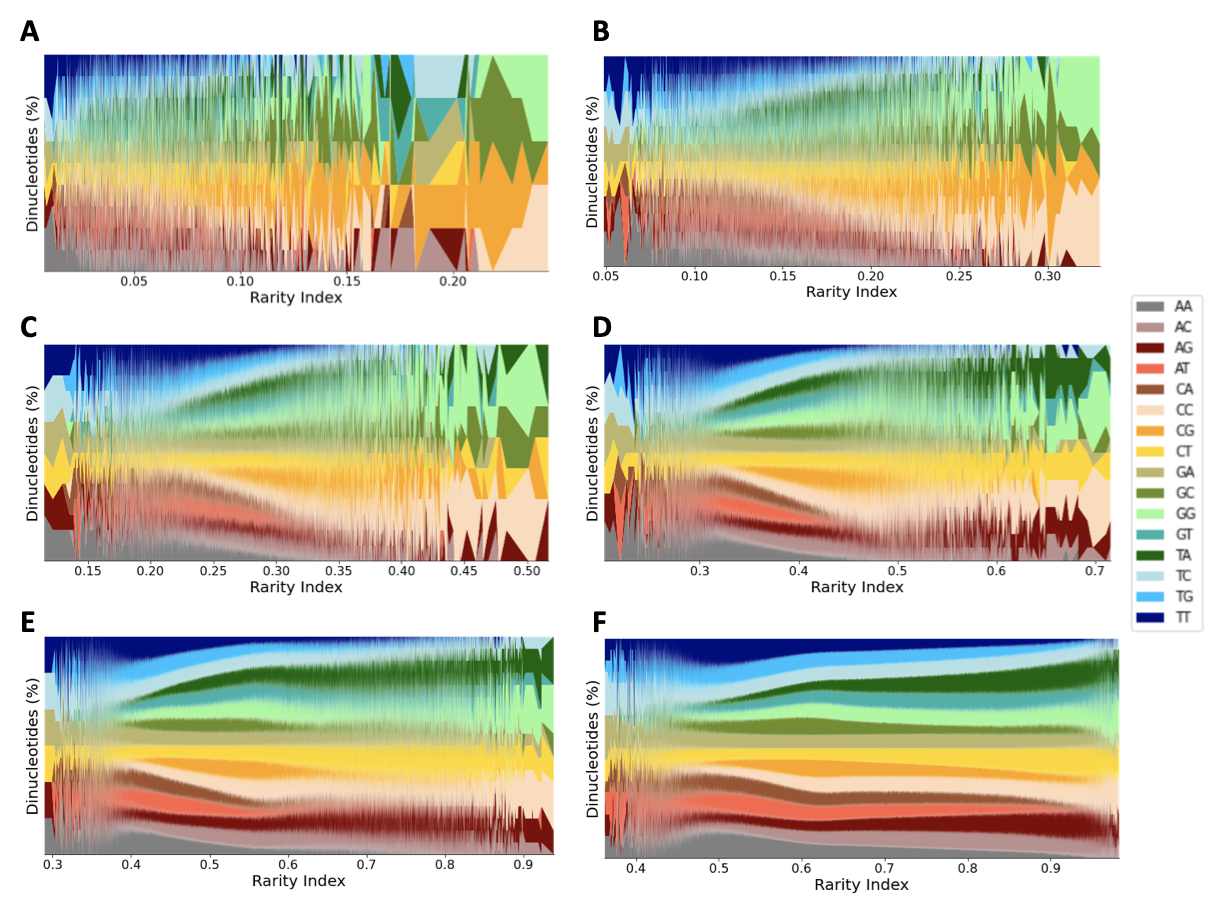


**Supplementary Figure 7: Association between the Rarity Index and dinucleotide content of kmers in genomes.** Results shown for: **A.** 6bp, **B.** 7bp, **C.** 8bp, **D.** 9bp, **E.** 10bp and **F.** 11bp.

**
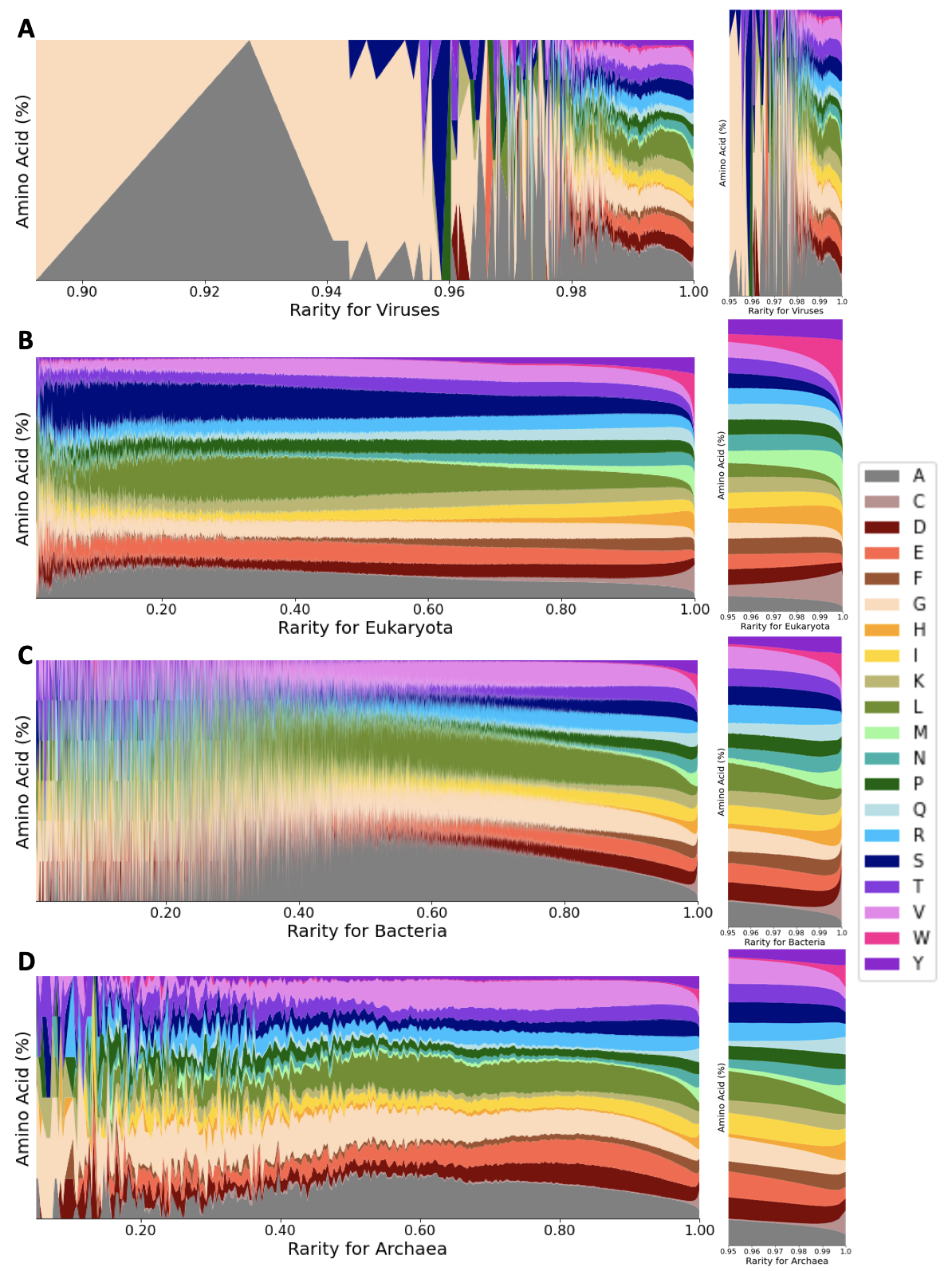
Supplementary Figure 8: Association between the Rarity Index and peptide content of kmers in proteomes.** Results shown for: **A.** Viruses, **B.** Eukaryotes, **C.** Bacteria and **D.** Archaea. Results shown for 6 amino acids kmer length.

**
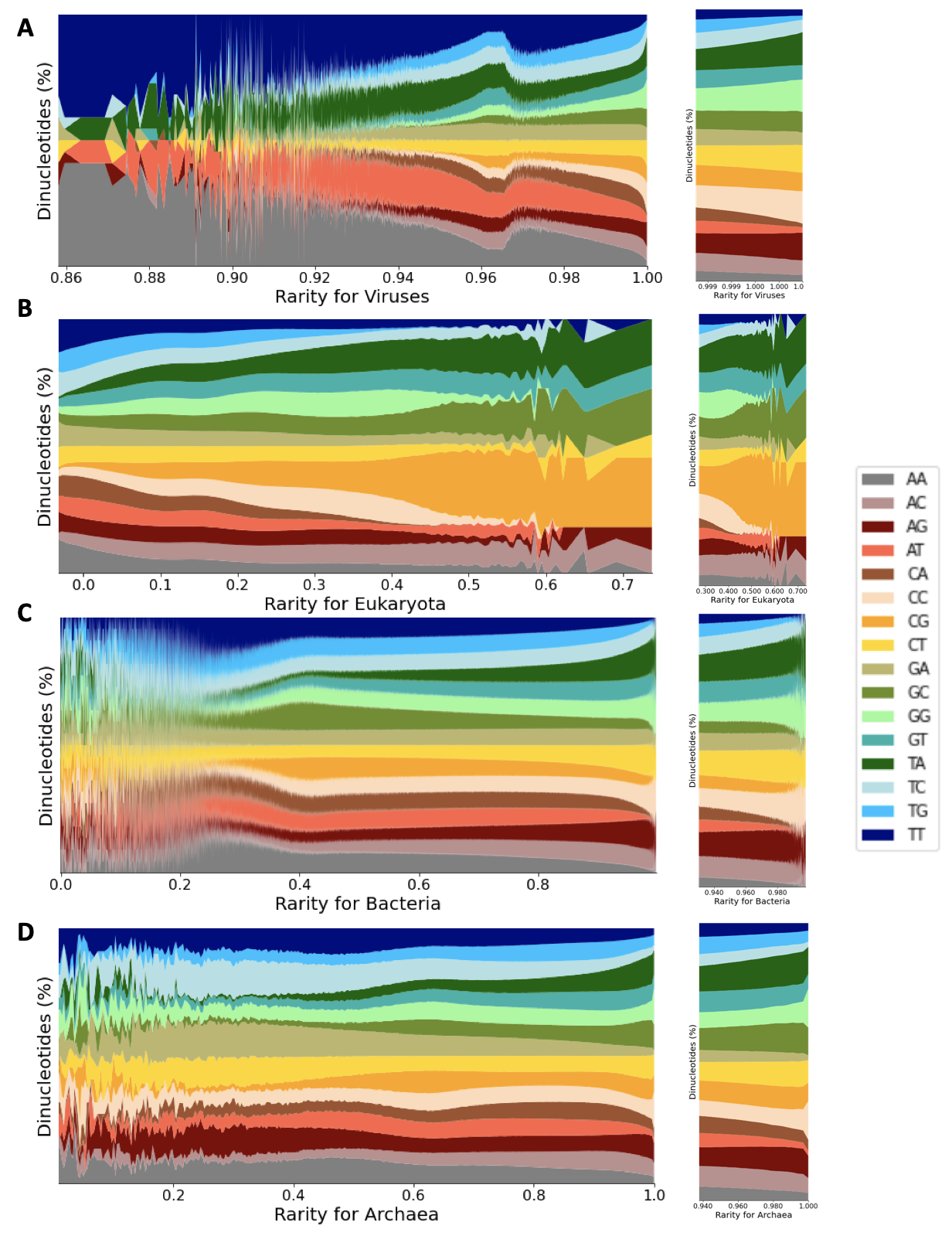
Supplementary Figure 9: Association between the Rarity Index and dinucleotide content of kmers in genomes.** Results shown for: **A.** Viruses, **B.** Eukaryotes, **C.** Bacteria and **D.** Archaea. Results shown for 12 bps kmer length.


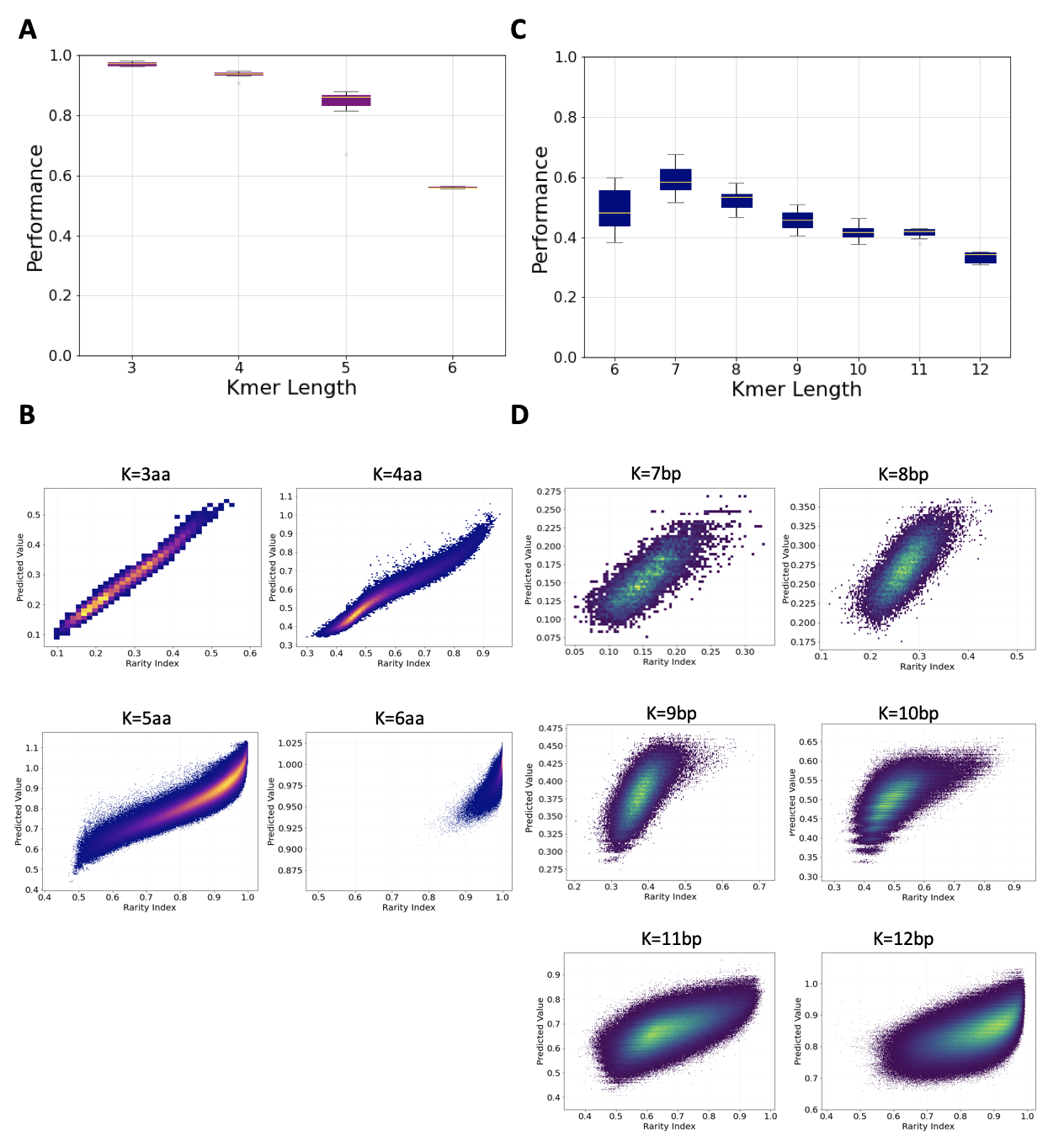


**Supplementary Figure 10: Predictive sequence-based models for the rarity of peptide and nucleic kmers across reference proteomes and genomes. A.** Performance of a ridge regression model for kmer lengths of six to twelve bp kmer lengths. **B.** Rarity index versus predicted value for each kmer for kmer lengths of three to six amino acids (aa). **C.** Performance of a ridge regression model for kmer lengths of six to twelve bp kmer lengths. **D.** Rarity index versus predicted value for each kmer for kmer lengths of seven to twelve bps.


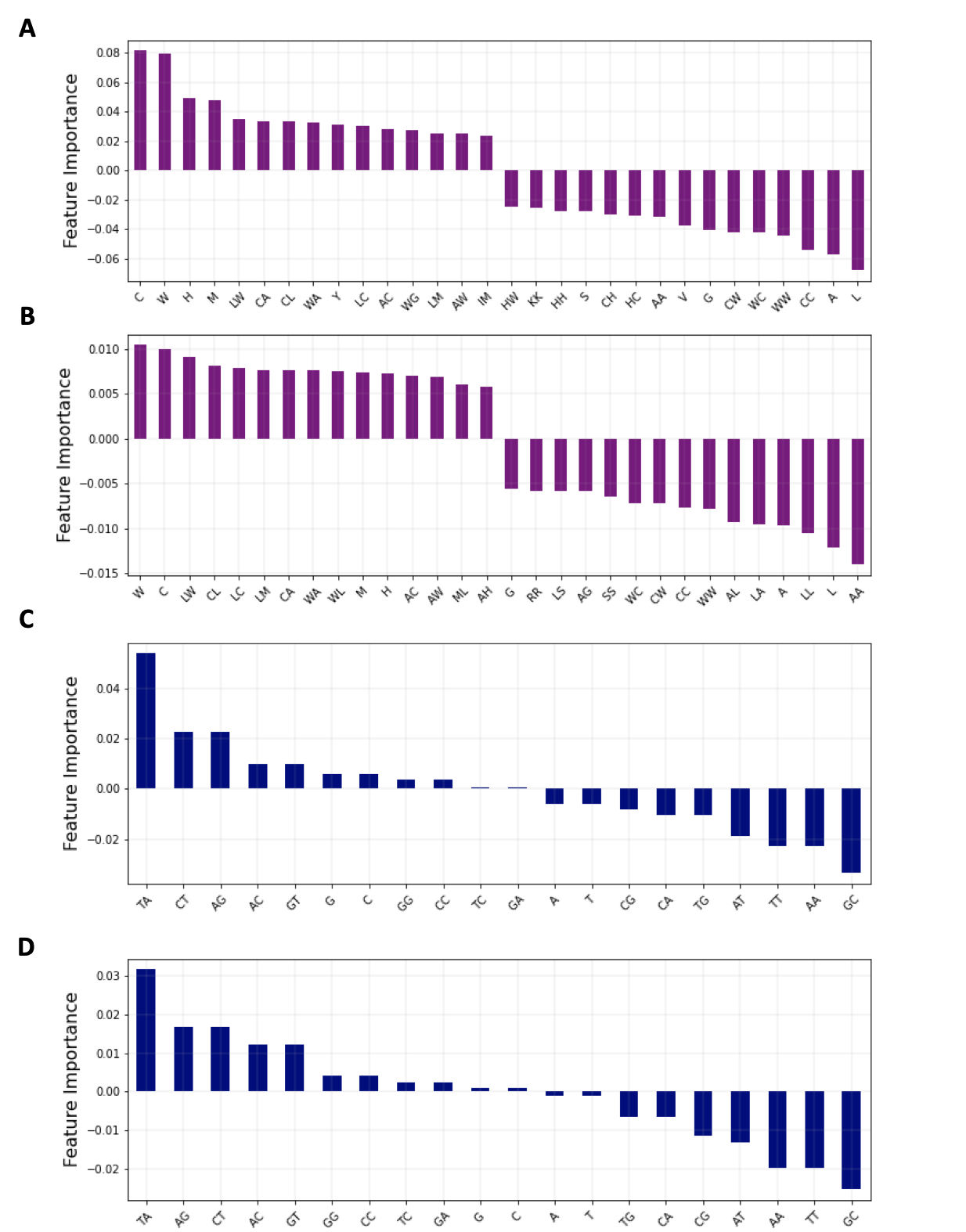


**Supplementary Figure 11: Feature importance for the coefficients of ridge regression models for peptide and kmer rarity.** Results shown for: **A.** 5 amino acid kmer length, **B.** 6 amino acid kmer length, **C.**11 bp kmer length, **D.**12 bp kmer length. The fifteen most positive and fifteen most negative coefficients are shown in A. and B.


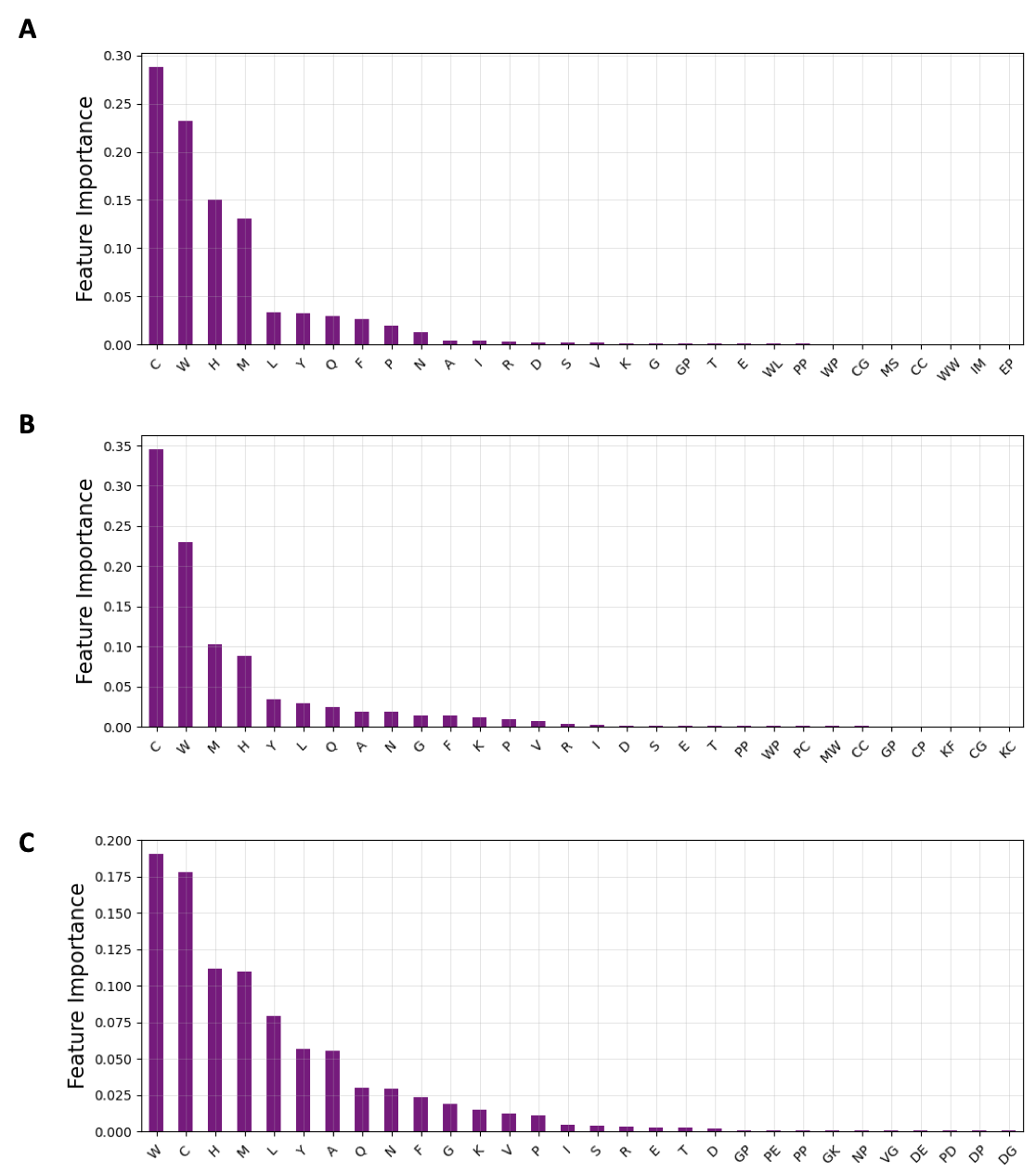


**Supplementary Figure 12: Feature importance for the coefficients of random forest regression models for peptide rarity.** Results shown for: **A.** 3 amino acid kmer length, **B.** 4 amino acid kmer length, **C.** 5 amino acid kmer length. The thirty most informative coefficients are shown in the panels.


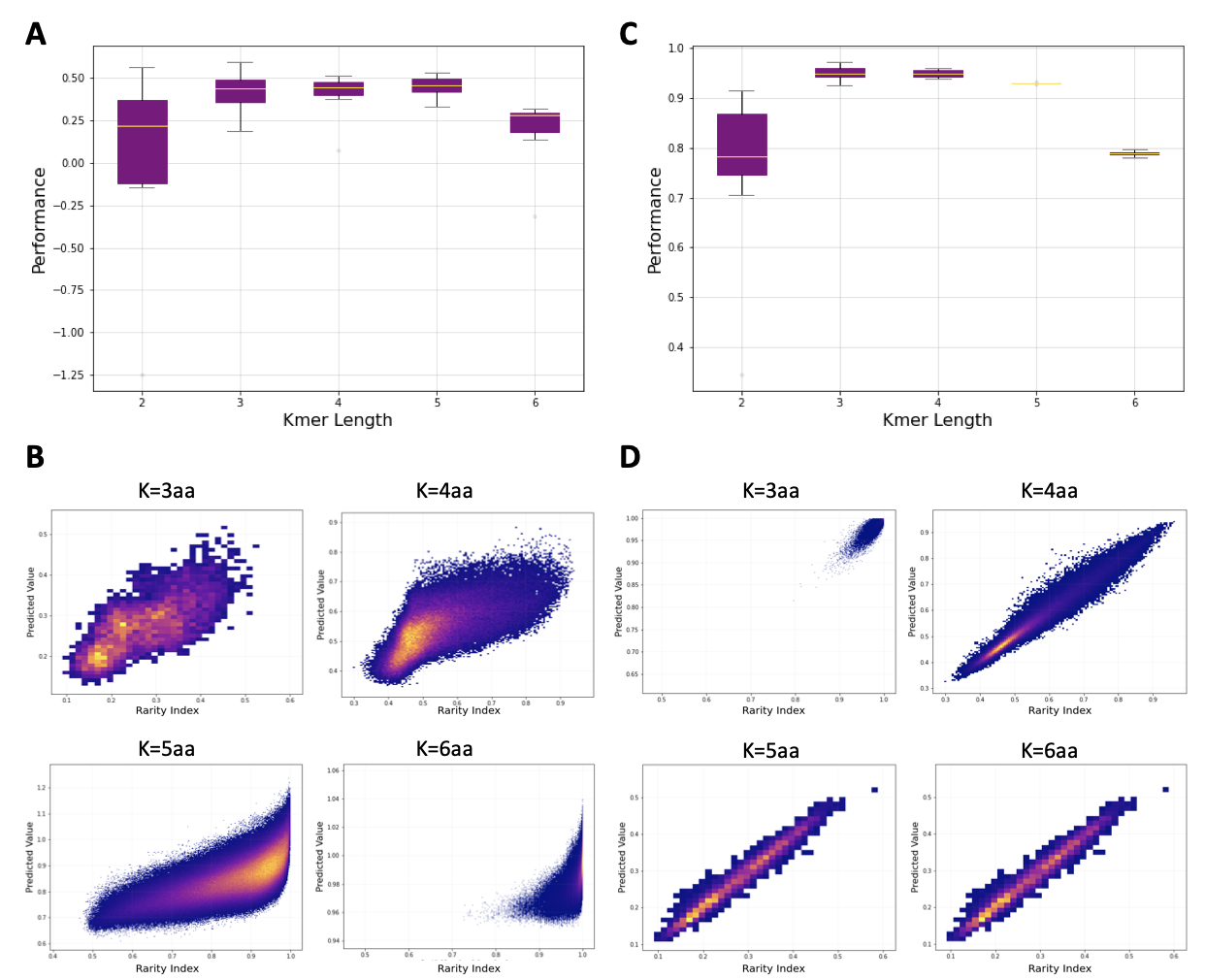


**Supplementary Figure 13: Predictive physicochemical-based models for the rarity of peptide kmers across organismal genomes. A.** Performance of a ridge regression model for peptide kmer lengths of three to six amino acids kmer lengths. **B.** Rarity index versus predicted value for each peptide kmer for kmer lengths of three to six amino acids kmer lengths using the ridge regression model. **C.** Performance of a random forest regression model for peptide kmer lengths of three to six amino acids kmer lengths. **D.** Rarity index versus predicted value for each peptide kmer for kmer lengths of three to six amino acids kmer lengths using the random forest regression model.


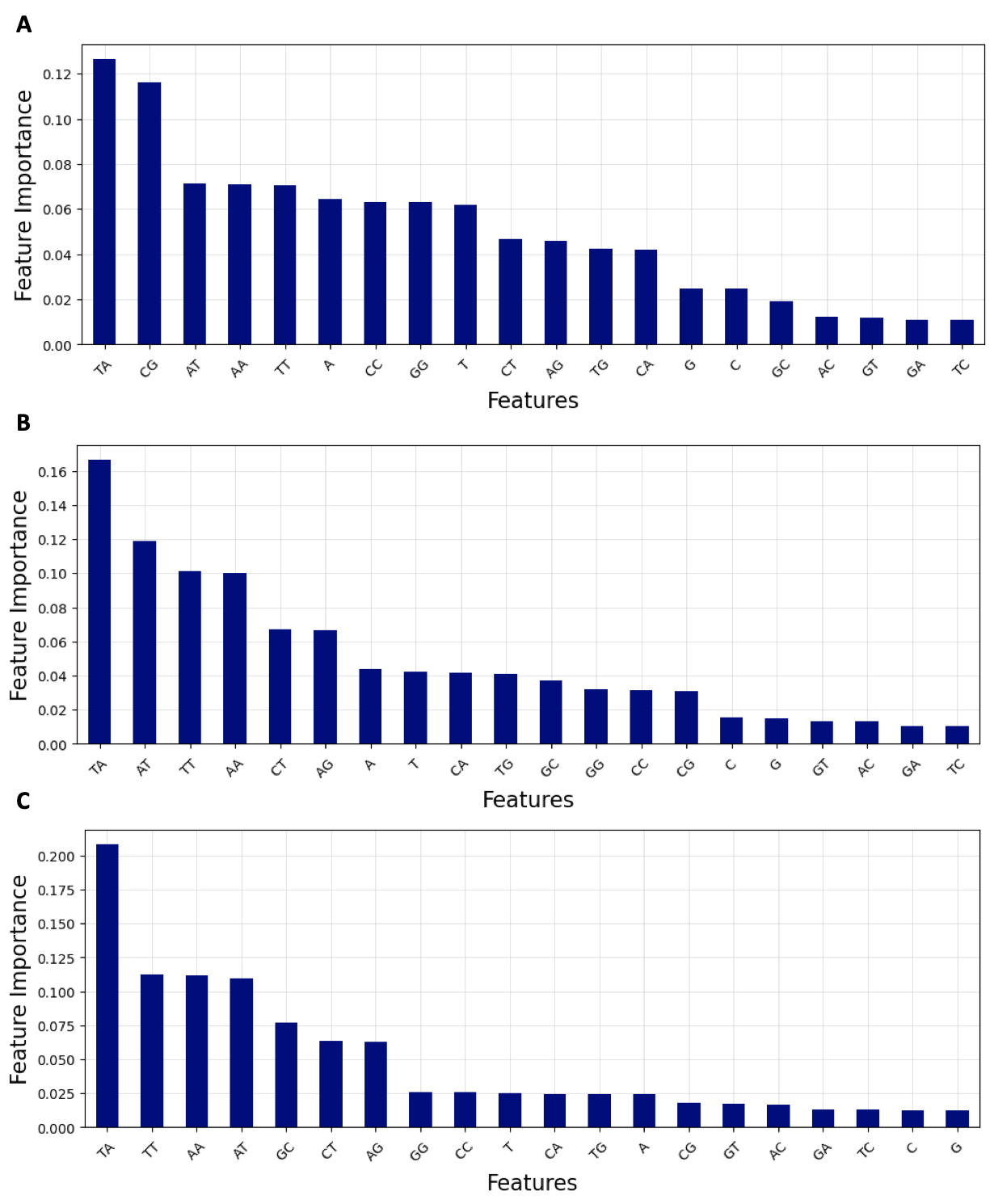


**Supplementary Figure 14: Feature importance for the coefficients of random forest regression models for nucleic rarity.** Results shown for: **A.** 9bp kmer length, **B.** 10bp kmer length, **C.** 11bp kmer length. The thirty most informative coefficients are shown in the panels.


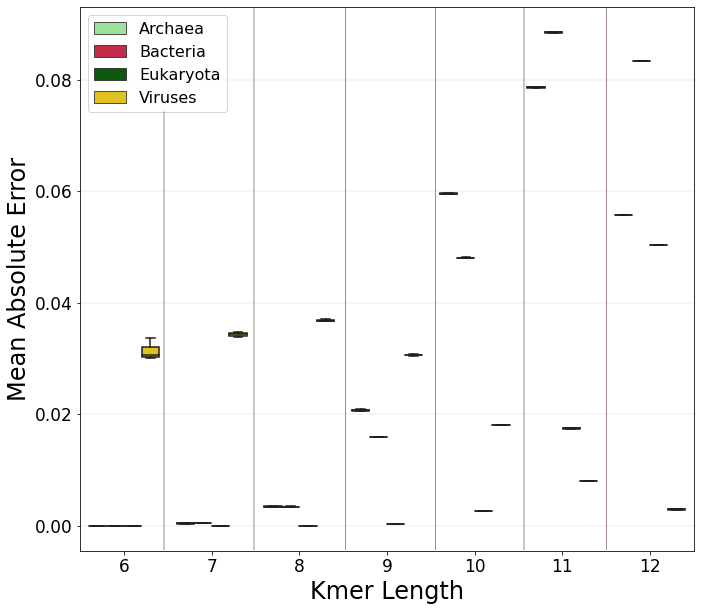


**Supplementary Figure 15: Mean Absolute Error for oligonucleotide sequence model predictions.**

**Supplementary Table 1: Rare peptide kmers in a single taxonomic group.** Peptide kmers that are below one standard deviation away from the mean of the Rarity Index in three of the four taxonomic groups (therefore are not rare in these taxonomies), and rarest in the fourth group. Only the top ten rarest kmers are shown per group.

| **Group** | **Kmer** | **Group** | **Kmer** | **Group** | **Kmer** | **Group** | **Kmer** |
| --- | --- | --- | --- | --- | --- | --- | --- |
| Viruses | GYGCTT | Eukaryota | VFQDPY | Archaea | ITPAHD | Bacteria | ECPGHF |
| Viruses | PWDGPA | Eukaryota | SGYPDC | Archaea | VAVYNH | Bacteria | MERDCL |
| Viruses | ILLTSM | Eukaryota | LMDEPC | Archaea | YHEAGH | Bacteria | RCFTCG |
| Viruses | PGDPEL | Eukaryota | GHTRWA | Archaea | SNPPYI | Bacteria | EMERDC |
| Viruses | GVQWHP | Eukaryota | TVYVTH | Archaea | RIDHYL | Bacteria | EMNLHV |
| Viruses | VIPQIS | Eukaryota | GYPDCR | Archaea | QDPYHG | Bacteria | HNQSPR |
| Viruses | VFIGSC | Eukaryota | LGEMNP | Archaea | YRIDHY | Bacteria | DGDEMN |
| Viruses | LKWPND | Eukaryota | GKYHPH | Archaea | DTTNIL | Bacteria | DEMNLH |
| Viruses | PWELGL | Eukaryota | TEIYDY | Archaea | IFSLEM | Bacteria | MNLHVP |
| Viruses | IHPGYG | Eukaryota | KYHPHG | Archaea | AENSNT | Bacteria | WVEKYR |

**Supplementary Table 2: Rare nucleic kmers in a single taxonomic group.** Nucleic kmers that are below one standard deviation away from the mean of the Rarity Index in three of the four taxonomic groups (therefore are not rare in these taxonomies), and rarest in the fourth group. Only the top ten rarest kmers are shown per group.

| **Group** | **Kmer** | **Group** | **Kmer** | **Group** | **Kmer** | **Group** | **Kmer** |
| --- | --- | --- | --- | --- | --- | --- | --- |
| Viruses | CCTTGTTACGAC | Eukaryota | TCGGGGGTTCGA | Archaea | GCTGCCGCCATT | Bacteria | CCTCCTCCTCTG |
| Viruses | AGTCGTAACAAG | Eukaryota | GGGGTTCGAATC | Archaea | GCTTGTGCAGCA | Bacteria | TCCTCCTCCTCT |
| Viruses | CTTTCCCTCACG | Eukaryota | TCATCGGCGGCG | Archaea | GCAGCTTGTTCA | Bacteria | TCCTTCCTTCTC |
| Viruses | AGTACGAGAGGA | Eukaryota | AGCGCCGCCGCC | Archaea | TGAACAAGCTGC | Bacteria | GTCCTCCTCCTT |
| Viruses | GCCGGCTTTTTC | Eukaryota | GGCGGCGGCGCT | Archaea | AAGCTGGTGATG | Bacteria | GAGGAGGAAGGA |
| Viruses | CATCCAGCTGTT | Eukaryota | TCCTGCTGGCGG | Archaea | CAATCTGCTGCT | Bacteria | CTGAAGAGGAGG |
| Viruses | ACGGGCGGTGTG | Eukaryota | ATCCGCCAGCAG | Archaea | CAGCATGATCAA | Bacteria | AGGAGAGGAGGA |
| Viruses | ACACACCGCCCG | Eukaryota | CTGCTGGCGGAT | Archaea | ATGATGACTTGA | Bacteria | ATGTACTCCTTC |
| Viruses | AAGTCGTAACAA | Eukaryota | ATTATTTAATAA | Archaea | CGCAAGAAAAAA | Bacteria | ATCATCCTCCTC |
| Viruses | TCGTAACAAGGT | Eukaryota | GGCTTCCGCTTC | Archaea | GCCACCACCAAA | Bacteria | TCGTCCTCCTCT |
